# Multivariate associations between neuroanatomy and cognition in unmedicated and medicated individuals with schizophrenia

**DOI:** 10.1038/s41537-024-00482-0

**Published:** 2024-07-14

**Authors:** Qiannan Zhao, Ziyang Gao, Wei Yu, Yuan Xiao, Na Hu, Xia Wei, Bo Tao, Fei Zhu, Siyi Li, Su Lui

**Affiliations:** 1 Department of Radiology, and Functional and Molecular Imaging Key Laboratory of Sichuan Province, West China Hospital of Sichuan University, Chengdu, China; 2 Huaxi MR Research Center (HMRRC), West China Hospital of Sichuan University, Chengdu, China; 3 Research Unit of Psychoradiology, Chinese Academy of Medical Sciences, Chengdu, China

**Keywords:** Schizophrenia, Biomarkers

## Abstract

Previous studies that focused on univariate correlations between neuroanatomy and cognition in schizophrenia identified some inconsistent findings. Moreover, antipsychotic medication may impact the brain-behavior profiles in affected individuals. It remains unclear whether unmedicated and medicated individuals with schizophrenia would share common neuroanatomy-cognition associations. Therefore, we aimed to investigate multivariate neuroanatomy-cognition relationships in both groups. A sample of 59 drug-naïve individuals with first-episode schizophrenia (FES) and a sample of 115 antipsychotic-treated individuals with schizophrenia were finally included. Multivariate modeling was conducted in the two patient samples between multiple cognitive domains and neuroanatomic features, such as cortical thickness (CT), cortical surface area (CSA), and subcortical volume (SV). We observed distinct multivariate correlational patterns between the two samples of individuals with schizophrenia. In the FES sample, better performance in token motor, symbol coding, and verbal fluency tests was associated with greater thalamic volumes but lower CT in the prefrontal and anterior cingulate cortices. Two significant multivariate correlations were identified in antipsychotic-treated individuals: 1) worse verbal memory performance was related to smaller volumes for the most subcortical structures and smaller CSA mainly in the temporal regions and inferior parietal lobule; 2) a lower symbol coding test score was correlated with smaller CSA in the right parahippocampal gyrus but greater volume in the right caudate. These multivariate patterns were sample-specific and not confounded by imaging quality, illness duration, antipsychotic dose, or psychopathological symptoms. Our findings may help to understand the neurobiological basis of cognitive impairments and the development of cognition-targeted interventions.

## Introduction

Impaired cognitive functioning is a major symptom of schizophrenia and is associated with poor prognosis and quality of life in affected individuals^1,2^. Considerable variability in cognition has been reported in individuals with schizophrenia across different illness courses and in individuals at high risk of psychosis^3–7^, which poses challenges for developing effective interventions. Additionally, previous research^8,9^ indicates that antipsychotics may impact the cognitive function of individuals with schizophrenia, emphasizing the complexity of discovering biomarkers for cognition-related therapy. Therefore, characterizing the neural substrates for cognitive impairments in schizophrenia is critical for developing cognition-targeted interventions and improving our understanding of the neurobiological mechanisms underlying impaired cognition.

Widespread brain morphological alterations defined by structural magnetic resonance imaging (MRI) have been robustly reported in individuals with schizophrenia^10–12^. These changes are accompanied by prominent heterogeneity^13–15^ and may contribute to cognitive impairments in schizophrenia. Clarifying cognition-related brain structural characteristics could help identify the neural basis for different cognitive profiles in schizophrenia.

Previous univariate correlational studies have identified diffuse neuroanatomy-cognition associations but achieved numerous inconsistent findings in schizophrenia^16,17^. This may be because these studies only looked at individual variables and ignored inter-neuroanatomy or inter-cognition associations. Multivariate correlational approaches have been proposed to overcome this limitation. A recent methodological study^18^ confirmed that multivariate modeling is superior to univariate models for brain-wide association studies. Adopting a multivariate approach may help better understand the complex relationship between brain structure and cognition in schizophrenia.

Multivariate correlational analyses have been used to link brain structure and behavior in both psychotic individuals^19–21^ and general populations^22,23^. However, while several studies have been conducted on cross-diagnostic individuals with psychiatry or healthy populations, very few have investigated the multivariate neuroanatomy-cognition patterns in individuals with schizophrenia. A previous study^24^ found that cognitive impairments, negative symptom severity, and brain abnormalities in default mode and visual networks were correlated in patients with schizophrenia. To better understand the neuroanatomy-cognition patterns, more studies directly targeted schizophrenia samples with multivariate designs are needed, particularly for patients at different illness stages or undergoing various treatments.

Schizophrenia is a progressive brain disorder that is associated with disparate patterns of neuroanatomic abnormalities at different illness stages^25,26^. Antipsychotic medications play a crucial role in brain structural changes over time^27^. Thus, the illness course and the exposure to antipsychotics may influence the relationships between neuroanatomic and cognitive profiles in this disorder. However, it remains to be elucidated whether unmedicated and medicated individuals with schizophrenia share common patterns of multivariate neuroanatomic-cognitive relationships.

Our current study aimed to investigate the multivariate relationships of neuroanatomic-cognitive profiles in individuals with schizophrenia who received antipsychotic medications or not. We included two independent patient samples, one composed of 59 drug-naïve individuals with first-episode schizophrenia (FES), and the other including 115 individuals with schizophrenia who were receiving antipsychotic medications. We conducted multivariate correlation analyses between multiple cognitive domains and neuroanatomic features, which involved regional measurements of cortical thickness (CT), cortical surface area (CSA), and subcortical volume (SV). Previous studies in general populations^28–31^ have suggested that CT and CSA may differ in developmental trajectories, genetic contributions, or relationships with cognitive function. Thus, we employed the combination of CT and SV or the combination of CSA and SV as neuroanatomic input for multivariate modeling in the two samples. We hypothesized that unmedicated and medicated individuals with schizophrenia would display distinct multivariate associations between neuroanatomic and cognitive profiles.

## Materials and methods

### Participants

This study included two samples with schizophrenia: 59 drug-naïve individuals with FES (illness duration: 20.07 [37.44] months) and 115 individuals with schizophrenia who were receiving antipsychotic treatment (illness duration: 229.26 [106.53] months). The FES sample consisted of community-dwelling individuals without exposure to antipsychotics or other psychopharmacological therapy. Medicated individuals with schizophrenia were maintained in the psychiatric institution, receiving ongoing atypical and (or) typical antipsychotic medications. The diagnosis of schizophrenia was based on the Structural Clinical Interview of Diagnostic and Statistical Manual for Mental Disorders (DSM-IV) (SCID). Positive and Negative Syndrome Scale (PANSS)^32^ and Global Assessment of Functioning (GAF)^33^ were used to evaluate psychopathological symptoms and functioning for affected individuals, respectively.

Demographically matched healthy controls were recruited from nearby communities, including 59 and 115 participants as normative references in brain-behavior profiles for the unmedicated and medicated samples, respectively. The non-patient version of SCID ensured that neither controls nor their relatives had current or historical psychiatric disorders. Exclusion criteria for all participants contained: a) age beyond the range of 18 to 65 years; b) a history of neuropathological diseases, head injuries, or systematic illness; c) a history of substance abuse; or d) MR contraindications such as claustrophobia, mental fixation, or pregnancy. This study was approved by the Ethics Committee on Biomedical Research, West China Hospital of Sichuan University and written informed consent was obtained from all participants.

### Structural MR imaging acquisition and preprocessing

Three-dimensional T1 weighted images of the head were acquired from the two data sets on 3-T MRI scanners at the Department of Radiology, West China Hospital of Sichuan University, Chengdu, China. For the FES sample and matched controls, images were acquired on a GE Signa EXCITE scanner using a spoiled gradient recalled (SPGR) sequence with an 8-channel head coil, and the following parameters were applied: repetition time (TR) = 8.5 ms, echo time (TE) = 3.4 ms, flip angle (FA) = 12°, 156 slices, matrix = 240 × 240 mm^2^, voxel size = 1 × 1 × 1 mm^3^. Images from antipsychotic-treated individuals and matched controls were acquired on a Siemens Trio scanner utilizing a magnetization-prepared rapid gradient-echo (MP-RAGE) sequence with a 32-channel head coil, following the Human Connectome Project (HCP) protocol (https://protocols.humanconnectome.org/), and the following parameters were used: TR = 2400 ms, TE = 2.01 ms, FA = 8°, 208 slices, matrix = 256 × 256 mm^2^, voxel size = 0.8 × 0.8 × 0.8 mm^3^.

The quality of all images had been inspected by an experienced neuroradiologist for the exclusion of participants with any gross abnormalities or scanning artifacts before being included in this study. The FreeSurfer (https://surfer.nmr.mgh.harvard.edu/) version 6.0 with the “recon-all” pipeline was applied to all the sMRI images for preprocessing. We then extracted CT and CSA from 68 cortical regions defined by the Desikan-Killiany atlas, volumes of 14 subcortical structures, total brain volume (TBV), and intracranial volume (ICV) for further analyses. Additionally, we calculated the Euler number, a metric for evaluating the quality of cortical reconstruction, based on the preprocessing results.

### Cognitive function evaluation

We used the Brief Assessment of Cognition in Schizophrenia (BACS)^34^ to evaluate the cognitive performance of individuals with schizophrenia and controls. The BACS consists of six test scores for different cognitive domains: verbal memory, verbal fluency, digit sequencing, token motor, symbol coding, and Tower of London test scores. The composite score is used to evaluate overall cognitive functioning.

### Multivariate analyses in brain-behavior dimensions

As a multi-to-multi approach, the canonical correlation analysis (CCA) method accounts for the complex between-feature relationships by maximizing the correlation between the linear combination of a set of item variables and the linear combination of another set of variables in a group of participants. The CCA algorithms are useful in detecting multivariate patterns that classical univariate approaches cannot yield, thus providing a deeper understanding of the brain-behavior associations. However, it is noteworthy that CCA algorithms may not perform well when the number of features exceeds the sample size.

The sparse CCA (sCCA) algorithm is a variant of the CCA method. It employs regularization to penalize item variables with low contributions to the model, which decreases the model complexity and the risks of overfitting. There are some useful terms when interpreting the results of the sCCA approach. First, the canonical variate or latent variate is the linear combination of item variables. Second, the canonical mode refers to each pair of canonical variates. Third, the canonical weights are the index for item variables composed of each pair of canonical variates. Item variables that are penalized weight zero. Each pair of canonical variates is composed of item variables with non-zero weights. Last, variable-to-variate univariate correlation is calculated to assess associations between identified latent variates and individual variables. Loadings are correlation coefficients for a certain variate with variables on the same side, while cross-loadings are correlation coefficients for a certain variate with variables on the opposite side.

**Fig. 1:**
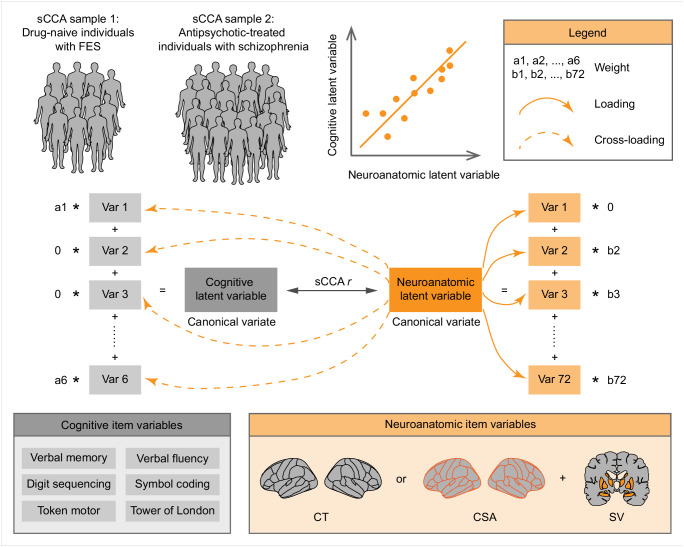
Schema of multivariate analyses between neuroanatomy and cognition in unmedicated and medicated individuals with schizophrenia. We conducted a series of sCCA between cognitive and neuroanatomic profiles in unmedicated and medicated individuals with schizophrenia. Six test scores from the BACS were included as cognitive item variables; Cortical measurements from 68 regions and volumes of 14 subcortical structures were included, and we used the combination of CT/SV or CSA/SV as neuroanatomic item variables. Item variables with low contributions to the model were penalized as zero, and non-zero weights were reported to demonstrate the composition of the identified canonical correlation. Loadings were defined as univariate correlation coefficients between one canonical variate and item variable on the same side; Cross-loadings were determined as univariate correlation coefficients between one canonical variate and item variable on the opposite side. BACS Brief Assessment of Cognition in Schizophrenia, FES first-episode schizophrenia, CSA cortical surface area, CT cortical thickness, sCCA sparse canonical correlation analysis, Var variable, sCCA r the coefficient for the significant canonical correlation, SV subcortical volume.

In this study, we used the R software (version 4.2.2) to conduct sCCA^22^ between cognitive and neuroanatomic features in the two samples of individuals with schizophrenia (see Fig. 1 for study design). We included six test scores of the BACS as cognitive item variables for multivariate modeling. It is important to note that the neurobiological implications represented by CT and CSA may differ. Furthermore, different developmental trajectories have been reported for CT and CSA in general populations^28,29^, as well as genetic contributions^30^, and relationships with cognitive profiles^31^. In our previous study, we also observed that CT and CSA measurements showed disparate abnormal patterns in antipsychotic-treated individuals with schizophrenia^35^. To achieve a balance between feature numbers and sample size, we used the combination of CT and SV or the combination of CSA and SV as neuroanatomic item variables to be analyzed in the multivariate modeling. We removed the variance related to covariates and performed standardization into z-scores for the input features of the sCCA (for details of covariates, see the section “Case-control analyses”). We used permutation tests to test the significance of sCCA results. For each identified canonical mode, we reported and visualized non-zero weights of item variables and cross-loadings with the threshold at ±0.20 or without the threshold.

### Test of specificity for canonical modes

We measured the specificity of identified canonical modes by testing their generalizing ability across two samples with schizophrenia. For this, canonical weights were extracted for a certain canonical mode identified in one sample and used to calculate predicted latent variables in the other sample. We conducted univariate Pearson correlation analyses between predicted latent variables for each identified mode. The specificity of a canonical mode in a certain sample was defined as the non-significant univariate correlation between predicted latent variables in the other sample. Additionally, we performed sCCA in control participants and further compared the neuroanatomy-cognition patterns between cases and controls.

### Sensitivity analysis of canonical modes

To determine whether the canonical modes identified in patients would be affected by various factors, such as imaging preprocessing quality (measured by the Euler number), illness duration, age at illness onset, daily dose of antipsychotics, or psychopathological symptoms (measured by the PANSS subset and total scores), we conducted separate univariate correlation analyses between canonical latent variables and each of these potential confounders.

### Cross-sample comparisons

We initially compared the demographic data, clinical information, and psychopathological symptoms of two samples with schizophrenia. Two-sample t-tests and Chi-squared tests were employed to detect between-sample differences in continuous and categorical variables, respectively. The false discovery rate (FDR) adjustments were applied to p-values for PANSS scores.

### Case-control analyses

Due to our data-driven design, no feature selection procedures were applied before the sCCA algorithm was performed. Thus, we investigated case-control differences in neuroanatomic and cognitive profiles for each cohort. Two-sample t-tests were conducted on residuals of cognitive or neuroanatomic measures after regressing out covariates. For comparisons in cognitive measures, age, sex, and educated years were as covariates; for tests of CSA and SV, age, sex, and ICV were included as uninterest variables; for detecting differences in CT, we included age and sex as covariates. The FDR adjustment was performed for case-control analyses across cognitive test scores and each type of neuroanatomic measure. To investigate the global neuroanatomic alterations of the two samples with schizophrenia, case-control comparisons in TBV and ICV were also conducted after the removal of variance related to age and sex.

## Results

### Cross-sample comparisons in demographics and clinical profiles

The antipsychotic-treated sample had significantly greater age, illness duration, percentage of males, and GAF score, but lower PANSS scores than the FES sample (Table 1). No significant between-sample differences in educational level were found.

**Table 1:** Cross-sample comparisons in demographics and clinical profiles for two samples of individuals with schizophrenia.

Measure	Drug-naïve FES sample (*N* = 59)	Antipsychotic-treated sample (*N* = 115)	t/χ^2^	*p*-value
Age (M [SD], years)	28.46 (9.24)	45.95 (7.10)	−12.73	**<0.001**
Sex (Female, N/N%)	31 (52.5%)	41 (35.7%)	4.59	**0.032**
Education level (M [SD], years)	11.05 (3.40)	10.15 (3.10)	1.71	0.090
Illness duration (M [SD], months)	20.07 (37.44)	229.26 (106.53)	−18.84	**<0.001**
Length of current hospitalization (M [SD], days)	NA	704.02 (1020.71)	NA	NA
Total length of hospitalization (M [SD], days)	NA	1433.49 (1016.71)	NA	NA
Number of lifetime hospitalization (M [SD])	NA	6.52 (6.24)	NA	NA
CPZ equivalent (M [SD], mg/day)	NA	559.20 (312.33)	NA	NA
Antipsychotic type (n/%)	NA	NA	NA	NA
Typical antipsychotics	NA	11 (9.6%)	NA	NA
Atypical antipsychotics	NA	79 (68.7%)	NA	NA
Mixed types of antipsychotics	NA	20 (17.4%)	NA	NA
Not available	NA	5 (4.3%)	NA	NA
PANSS (M [SD])	NA	NA	NA	NA
Positive score	24.93 (3.61)	10.61 (4.74)	22.06	**<0.001**
Negative score	23.22 (4.71)	16.04 (5.45)	8.97	**<0.001**
General psychopathological score	48.32 (6.68)	25.79 (6.07)	21.61	**<0.001**
Total score	96.47 (11.39)	52.44 (13.88)	22.25	**<0.001**
GAF (M [SD])	50.25 (15.24)	59.53 (14.85)	−3.82	**<0.001**

The *p* values generated for between-sample comparisons of PANSS scores were corrected by false discovery rate (FDR). Boldface indicates *p* < 0.05.

*CPZ* chlorpromazine equivalent used to estimate the daily dose of antipsychotics, *GAF* Global Assessment of Functioning, *M* mean value, *PANSS* Positive and Negative Syndrome Scale, *SD* standard deviation, *t* t statistic in the two-sample t-test, *χ*^2^ chi-square statistic.

### Multivariate neuroanatomic-cognitive patterns in two patient samples

**Fig. 2:**
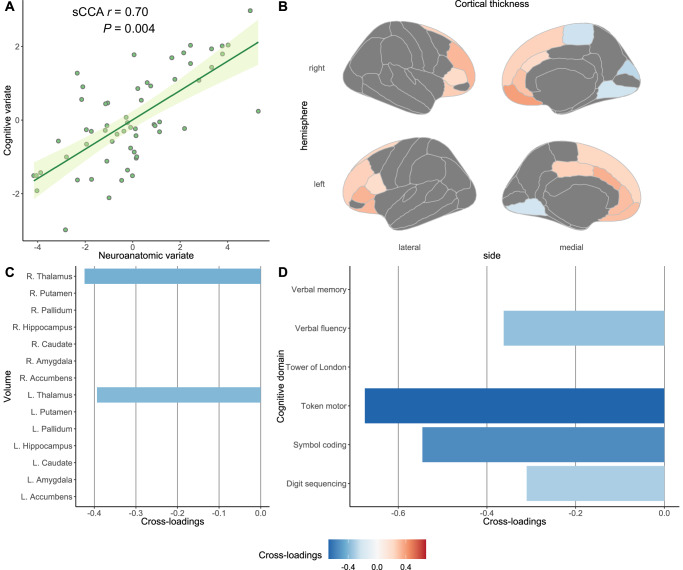
A significant canonical mode between brain-behavior profiles and corresponding cross-loadings with the threshold at ± 0.20 in the FES sample. **A** We identified a significant canonical mode between CT/SV neuroanatomic features and cognitive domains in drug-naïve individuals with FES using an sCCA algorithm. Cross-loadings, representing univariate correlation coefficients between each latent variate and the opposite item variables, were extracted at a threshold of ±0.20. We demonstrate cross-loadings between (**B**) the cognitive variate and CT item variables, (**C**) between the cognitive variate and SV item variables, and (**D**) between the neuroanatomic variate and cognitive item variables, for the significant canonical mode in FES individuals. CT cortical thickness, L the left hemisphere, FES first-episode with schizophrenia, *p* *p*-value generated in the permutation test, *R* the right hemisphere, *sCCA* sparse canonical correlation analysis, *sCCA r* the coefficient for the significant canonical correlation, *SV* subcortical volume.

**Fig. 3:**
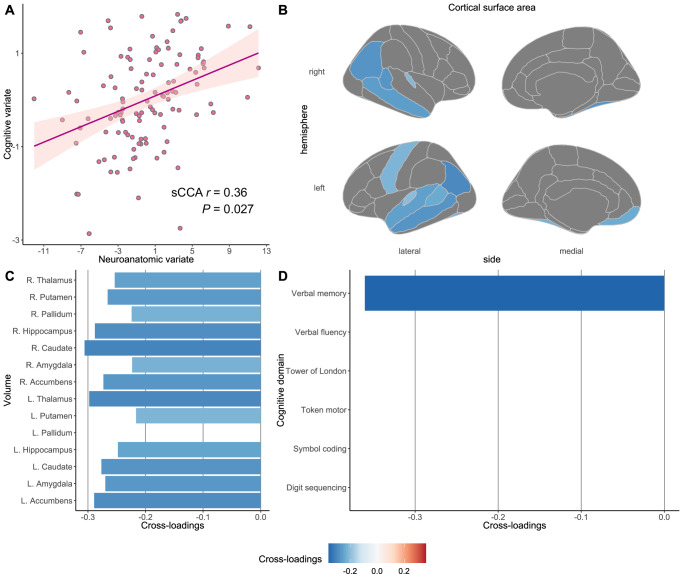
The first significant canonical mode (LV-1) between brain-behavior profiles and corresponding cross-loadings with the threshold at ±0.20 in the antipsychotic-treated sample. **A** We identified two significant canonical modes between CSA/SV neuroanatomic features and cognitive domains in antipsychotic-treated individuals with schizophrenia using an sCCA algorithm and demonstrated the first one in this figure. Cross-loadings, representing univariate correlation coefficients between each latent variate and the opposite item variables, were extracted at a threshold of ±0.20. We demonstrate cross-loadings between (**B**) the cognitive variate and CSA item variables, (**C**) between the cognitive variate and SV item variables, and (**D**) between the neuroanatomic variate and cognitive item variables, for the first significant canonical mode in antipsychotic-treated individuals with schizophrenia. *CSA* cortical surface area, *L* the left hemisphere, *p* *p*-value generated in permutation tests, *R* the right hemisphere, *sCCA* sparse canonical correlation analysis, *sCCA r* the coefficient for the first significant canonical correlation, *SV* subcortical volume.

**Fig. 4:**
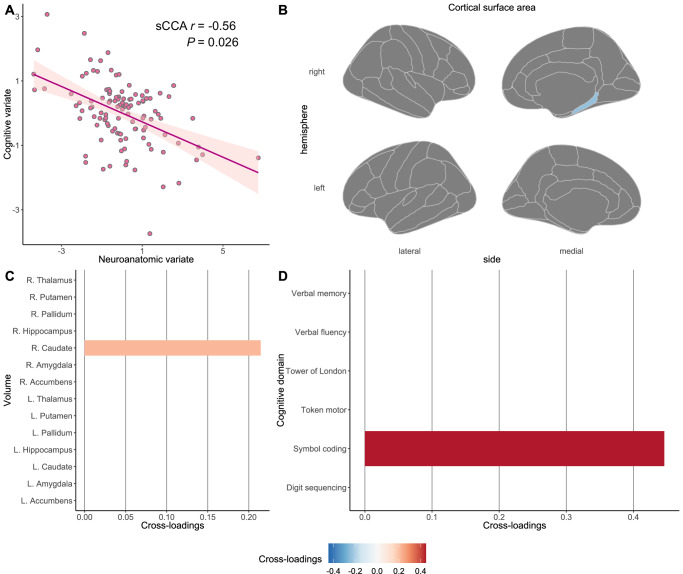
The second significant canonical mode (LV-2) between brain-behavior profiles and corresponding cross-loadings with the threshold at ±0.20 in the antipsychotic-treated sample. **A** We identified two significant canonical modes between CSA/SV neuroanatomic features and cognitive domains in antipsychotic-treated individuals with schizophrenia using an sCCA algorithm and demonstrated the second one in this figure. Cross-loadings, representing univariate correlation coefficients between each latent variate and the opposite item variables, were extracted at a threshold of ±0.20. We demonstrate cross-loadings between (**B**) the cognitive variate and CSA item variables, (**C**) between the cognitive variate and SV item variables, and (**D**) between the neuroanatomic variate and cognitive item variables, for the second significant canonical mode in antipsychotic-treated individuals with schizophrenia. *CSA* cortical surface area, *L* the left hemisphere, *p* *p*-value generated in permutation tests, *R* the right hemisphere, *sCCA* sparse canonical correlation analysis, *sCCA r* the coefficient for the significant canonical correlation, *SV* subcortical volume.

A series of sCCA analyses identified disparate multivariate associations between neuroanatomic and cognitive profiles in two independent samples with schizophrenia. This result involved different types of neuroanatomic measurement and various cognitive domains. In the FES sample, a canonical mode between CT/SV measures and multiple cognitive domains was retained (sCCA *r* = 0.70, *p* = 0.004) (Fig. 2). Conversely, two significant canonical modes were observed in antipsychotic-treated patients between CSA/SV measures and functioning of the single cognitive domain (LV-1: sCCA *r* = 0.36, *p* = 0.027; LV-2, sCCA *r* = −0.56, *p* = 0.026) (Figs. 3 and 4). For each pair of latent variates, features with low contributions were penalized and non-zero weights could be found in Supplementary Figs. S1–S3. Cross-loadings with a threshold at ±20 or without a threshold for each identified canonical mode could be found in Figs. 2–4 and Supplementary Figs. S4–S6, respectively.

### The canonical mode in the drug-naïve FES sample

In the FES sample, the latent cognitive variate, comprised of token motor (weight = −0.84), symbol coding (weight = −0.53), and verbal fluency (weight = −0.10) test scores (Supplementary Fig. S1), displayed the higher positive cross-loadings with CT in the right medial orbitofrontal cortex (cross-loading = 0.39), the left caudal anterior cingulate cortex (cross-loading = 0.34), the left pars triangularis (cross-loading = 0.33), and the right rostral middle frontal gyrus (cross-loading = 0.33) and highest negative cross-loadings with thalamus volumes (cross-loading for the left thalamus = −0.39; cross-loading for the right thalamus = −0.42) (Fig. 2 and Supplementary Fig. S4). This significant canonical mode indicates that better cognitive performance for multiple domains was associated with greater thalamic volumes but thinner cortices in the prefrontal regions and anterior cingulate cortex.

### Canonical modes in the antipsychotic-treated sample

#### Latent-varaible-1 (LV-1)

For the first significant mode in antipsychotic-treated patients, the latent cognitive variate only represented verbal memory test scores (weight = −1.00) (Supplementary Fig. S2). This variate had the highest negative cross-loadings with volumes in the right caudate (cross-loading = −0.31), the left thalamus (cross-loading = −0.30), the left nucleus accumbens (cross-loading = −0.29), and the right hippocampus (cross-loading = −0.29), as well as with CSA in the left inferior parietal cortex (cross-loading = −0.30) (Fig. 3 and Supplementary Fig. S5). The LV-1 identified in antipsychotic-treated individuals with schizophrenia suggests poor verbal memory performance was related to smaller volumes for the most subcortical structures and smaller CSA, mainly in temporal cortices and inferior parietal lobule.

#### LV-2

In terms of the second significant mode in medicated patients, the latent cognitive variate was composed of symbol coding test scores (weight = −1.00) (Supplementary Fig. S3), merely demonstrating a positive cross-loading with the right caudate volume (cross-loading = 0.21) and a negative cross-loading with CSA in the right parahippocampal gyrus (cross-loading = −0.24) at the threshold of ± 0.20 (Fig. 4 and Supplementary Fig. S6). Based on the LV-2 from the medicated sample, a lower symbol coding test score was associated with smaller CSA in the right parahippocampal gyrus but greater volume in the right caudate.

### Specificity for canonical modes

In antipsychotic-treated individuals with schizophrenia, the predicted latent variables, composed of CT/SV and cognitive measures, did not significantly correlate with each other (*r* = 0.06, *p* = 0.533) (Supplementary Fig. S7). Regarding generalizing two canonical modes between CSA/SV and cognitive measures defined by the antipsychotic-treated sample to the drug-naive sample, no significant correlations were identified between the predicted latent variables (predicted LV-1: *r* = 0.05, *p* = 0.729; predicted LV-2: *r* = -0.25, *p* = 0.056) (Supplementary Fig. S7). Additionally, the canonical modes identified in control participants differed from those in patients (Supplemental Results and Figs. S8–S14). These findings confirm the specificity of canonical modes identified in each schizophrenia sample.

### Sensitivity analysis of canonical modes

No significant correlations survived FDR corrections were found between canonical latent variables and confounding factors such as imaging quality index, illness duration, age at onset, daily antipsychotic dose, or psychopathological symptoms (Supplementary Tables S1 and S2).

### Case-control analyses

The FES sample was older and had a greater percentage of males and shorter educated years than matched controls (Supplementary Table S3). No significant demographical differences were observed between antipsychotic-treated patients and matched controls (Supplementary Table S3). Case-control results in regional CT, CSA, SV measures, and cognitive scores can be found in Supplemental Results and Figs. S15–S16 in Supplementary Material. Overall, near-normal neuroanatomic profiles were found in drug-naïve individuals with FES, except cortical thinning in the left fusiform gyrus (*t* = −3.93, *p*_uncorrected_ < 0.001, *p*_FDR_ = 0.010) and bilateral precentral gyri (left: *t* = −3.31, *p*_uncorrected_ = 0.001, *p*_FDR_ = 0.029; right: *t* = −3.30, *p*_uncorrected_ = 0.001, *p*_FDR_ = 0.029) as well as reductions on TBV (*t* = −3.59, *p*_FDR_ = 0.001) and ICV (*t* = −2.59, *p*_FDR_ = 0.011) relative to controls. Antipsychotic-treated patients displayed widespread gray matter reductions involving CT, CSA, and SV measures, where deficits were prominent in frontotemporal and subcortical regions. Conversely, increased volumes in the right pallidum and thicker cortex in the left caudal anterior cingulate gyrus were also observed in antipsychotic-treated patients.

## Discussion

The current study identified distinct multivariate patterns between cognitive and neuroanatomic profiles in unmedicated and medicated individuals with schizophrenia. Specifically, CT/SV and CSA/SV measurements were involved in unmedicated and medicated samples, respectively. In drug-naïve individuals with FES, better performance in multiple cognitive domains evaluated by token motor, symbol coding, and verbal fluency tests was associated with greater thalamic volumes but lower CT in the prefrontal regions and the anterior cingulate cortex. In antipsychotic-treated individuals with schizophrenia, two significant canonical modes were determined, each of which involves a single cognitive domain: (a) In LV-1, the worse verbal memory performance was related to smaller volumes for the most subcortical structures and smaller CSA predominantly in temporal regions and inferior parietal lobule; (b) In LV-2, the lower symbol coding test scores were associated with smaller CSA in the right parahippocampal gyrus but greater volumes in the right caudate. Moreover, these identified canonical modes were specific to each patient sample and not affected by confounding factors such as reconstructive quality of images, illness duration, antipsychotic dose, or psychopathological symptoms. These findings may facilitate the understanding of the neurobiological basis of cognitive impairments in schizophrenia and the discovery of biomarkers for cognition-targeted interventions.

Our main finding is the distinct multivariate relationships between neuroanatomic and cognitive function in the drug-naïve FES sample and the antipsychotic-treated group, involving CT/SV measurements with multiple cognitive domains and the CSA/SV measurements with two single cognitive domains, respectively. The differences between CT and CSA involvements in two samples are supported by evidence from general populations that these two neuroanatomic measurements have different associations with cognitive aspects and the associations vary across different ages^31,36^; for example, greater CSA in regions related to working memory, attention, and visuospatial preprocessing was associated with better performance in fluid intelligence while lower CT but greater CSA in language-related networks was related to greater crystallized intelligence scores. Our identification of distinct multivariate neuroanatomy-cognition patterns in two samples with schizophrenia may facilitate the understanding of the neurobiological basis for cognitive impairments with considerable variability. Antipsychotic treatment and illness courses may participate in disparate multivariate patterns revealed by the two samples and further investigation of potential mechanisms is needed with follow-up data from drug-naïve individuals with schizophrenia and animal models.

The significant canonical mode identified in drug-naïve individuals with FES captured multiple cognitive domains assessed by token motor, symbol coding, and verbal fluency tests; and better cognitive performance was associated with greater thalamic volumes but lower CT in the prefrontal regions and the anterior cingulate cortex. The token motor test measures motor speed, while the verbal fluency test assesses the ability of categorical and semantic fluency^34^. In contrast, the symbol coding test measures several cognitive domains, such as attention, processing speed, working memory, visuoperceptual function, and motor speed^37^. The cortical regions involved in this canonical mode, such as prefrontal regions and anterior cingulate cortices, play considerable roles in the cognitive domains mentioned above according to functional MR studies of FES^38–42^. Our findings of the negative CT-cognition associations observed in FES are broadly in line with results from univariate correlational studies of psychotic individuals at early illness stages, where frontoparietal regions are affected. For example, the worse attention performance was related to thicker cortices in the parietal lobe in first-episode psychosis (FEP)^43^; The relationships between worse verbal fluency performance and thicker cortices in the left intraparietal sulcus, the right precentral and fusiform gyri were reported in a group of young patients with psychosis^44^. Our finding indicates that cortical thinning is not always bad for cognition in schizophrenia. This notion is also supported by findings from general populations that cortical thickness in frontoparietal regions was negatively associated with crystal intelligence, a knowledge-based cognitive measure^31^.

Our finding in individuals with FES also revealed that smaller thalamus volumes were associated with worse performance in multiple cognitive domains. The thalamus is a hub for brain function and is crucial for cognitive functioning. Functional and structural alterations of the thalamus in schizophrenia have been reported and associated with cognitive impairments^45–47^. Our finding of the thalamus is consistent with univariate correlational results in FES individuals; For example, thalamic volume deficits were associated with worse performance in attention, language, motor, and executive functioning^48,49^. Additionally, thalamic shape compression in FES was associated with worse performance in executive functioning and working memory^50^.

In LV-1 identified in antipsychotic-treated individuals with schizophrenia, the worse verbal memory performance was associated with volume deficits in most subcortical regions and CSA deficits predominantly in temporal cortices and inferior parietal lobule. The positive relationships between volumes in the hippocampus and nucleus accumbens and verbal memory/learning test scores have been reported in patients with schizophrenia^51–55^. There have been fewer univariate correlational studies across schizophrenia spectrum disorders that discovered significant CSA-cognition relations relative to constructs involved in measures such as CT or volumes^17^. Replication of our results in studies with larger sample sizes is needed to confirm such multivariate patterns.

In LV-2 defined in antipsychotic-treated individuals with schizophrenia, the worse symbol coding performance was related to greater volumes in the right caudate but smaller CSA in the right parahippocampal gyrus. In a longitudinal study of individuals with schizophrenia, changes in caudate volumes were associated with cognitive domains, including attention, problem-solving, and working memory^56^, supporting the role of caudate in this canonical mode. An interesting finding in LV-2 is the relationship between the right parahippocampal CSA and symbol coding test scores, and this result indicates that greater CSA in the parahippocampal region may benefit symbol coding performance. Inconsistent findings have been identified in previous studies. In a cross-diagnostic study^19^ that captured structural-cognitive CCA modes in psychosis, better general cognitive function was related to larger volumes, and thicker cortices but smaller CSA in the mostly frontal-parietal regions, indicating that greater CSA is not always better for cognitive function. An inverse relationship between the right parahippocampal volume and other cognitive domains such as verbal intelligence, has been reported in men with schizophrenia using a univariate approach^57^.

Several limitations should be considered when interpreting the results of this study. First, different MRI scanners and acquisition parameters applied to the two patient samples may confound the distinct brain-behavior patterns that we reported. Especially for this fact, it is difficult to completely exclude the potential influence of scanning factors although no significant effects of cortical reconstruction quality, measured by the Euler number, on those identified latent variates, were identified. However, a recent study^58^ also confirms the great consistency (i.e., intraclass correlation coefficients range from 0.859 to 0.986 for cortical and subcortical measures) of FreeSurfer-derived measurements concerning different scanners or scanning sequences, enhancing the confidence for the reliability of our findings to some extent. Second, our cross-sectional design cannot explore the mechanisms for canonical modes in samples with schizophrenia. Third, slightly mismatched demographics between individuals with FES and corresponding control participants may bias the case-control results in brain-behavior profiles, although we had adjusted covariates in comparisons. Last, our sample sizes for the two cohorts with schizophrenia are relatively small, which may affect the power of capturing canonical modes, although significant canonical modes were identified in the two samples.

## Conclusions

In sum, we determined distinct multivariate patterns between cognitive and neuroanatomic profiles in unmedicated and medicated individuals with schizophrenia. These findings may facilitate the understanding of the neurobiological basis of cognitive impairments and the development of cognition-targeted interventions.

### Supplementary information

Supplementary Materials

## Data Availability

We do not have permission to share the data used in this study with the public.

## Code availability

The codes used to conduct sCCA can be found in this repository: https://github.com/AmirhosseinModabbernia/IMAGEN. No other customized codes were applied in this study.
