# Supplementary material for: Multivariate associations between neuroanatomy and cognition in unmedicated and medicated individuals with schizophrenia
Source: Schizophrenia (Heidelb). 2024 Jul 14;10(1):62. doi: 10.1038/s41537-024-00482-0 (PMC11247086; doi:10.1038/s41537-024-00482-0)
Supplement: Supplementary file 1 — Supplementary Materials [file 41537_2024_482_MOESM1_ESM.pdf]

Supplementary Material

Multivariate Associations between Neuroanatomy and Cognition in Unmedicated and Medicated Individuals with Schizophrenia

Zhao et al.

Table of Contents

Supplemental Results 3
Multivariate neuroanatomic-cognitive patterns in control participants 3
Case-control analyses of neuroanatomy-cognition features in the two data sets 3
Supplemental Figures 5
Figure S1. Non-zero weights of item variables for the significant pairs of latent variates identified in the drug-naïve sample with FES 5
Figure S2. Non-zero weights of item variables for the first significant pairs of latent variates (LV-1) identified in the antipsychotic-treated sample 6
Figure S3. Non-zero weights of item variables for the second significant pairs of latent variates (LV-2) identified in the antipsychotic-treated sample 7
Figure S4. Cross-loadings for the significant brain-behavior canonical mode in the drug-naïve FES sample without the restriction of threshold 8
Figure S5. Cross-loadings for the first significant brain-behavior canonical mode in the antipsychotic-treated sample without the restriction of threshold 9
Figure S6. Cross-loadings for the second significant brain-behavior canonical mode in the antipsychotic-treated sample without the restriction of threshold 10
Figure S7. Test of specificity for the identified canonical modes in two samples with schizophrenia 11
Figure S8. The first significant canonical mode (LV-S1) between brain-behavior profiles and corresponding cross-loadings with the threshold at ±0.20 in the control participants paired with the FES sample 12
Figure S9. Cross-loadings for the first significant brain-behavior canonical mode in the control participants paired with the FES sample without the restriction of threshold 13
Figure S10. Non-zero weights of item variables for the first significant pairs of latent variates (LV-S1) identified in the control participants paired with the FES sample 14
Figure S11. The second significant canonical mode (LV-S2) between brain-behavior profiles and corresponding cross-loadings with the threshold at ±0.20 in the control participants paired with the FES sample 15
Figure S12. Cross-loadings for the second significant brain-behavior canonical mode (LV-S2) in the control participants paired with the FES sample without the restriction of threshold 16
Figure S13. Non-zero weights of item variables for the second significant pairs of latent variates (LV-S2) identified in the control participants paired with the FES sample 17
Figure S14. Test of specificity for the identified canonical modes in FES patients and matched control participants 18
.... 19
Figure S15. Cortical maps for case-control comparisons in the two data sets 19
Figure S16. Case-control comparisons in SV and cognitive function in the two data sets . 20
Supplemental Tables 21
Table S1. Univariate correlations between neuroanatomic latent variables and demographics or clinical profiles in two samples with schizophrenia 21
Table S2. Univariate correlations between cognitive latent variables and demographics or clinical profiles in samples with schizophrenia 22
Table S3. Case-control comparisons in demographics within the data set 23

Supplemental Results

Multivariate neuroanatomic-cognitive patterns in control participants

Two significant canonical modes were identified in control participants paired with the FES sample, and no significant canonical modes were found in control participants paired with the antipsychotic-treated sample with schizophrenia.

The first significant canonical mode in control participants paired with the FES sample was identified between CT/SV neuroanatomic features and cognitive domains (LV-S1: sCCA r=0.72, p=0.006) (**Figure S8**). In LV-S1, the latent cognitive variate, comprised of Tower of London test scores (weight=-0.95), verbal memory test scores (weight=-0.25), verbal fluency test scores (weight=0.18), and token motor test scores (weight=-0.10), displayed the higher positive cross-loadings in the right pars orbitalis (cross-loading=0.34) and the right isthmus cingulate cortex (cross-loading=0.29), and highest negative cross-loadings with the left posterior cingulate cortex (cross-loading=-0.24) (**Figure S8 – S10**).

The second significant canonical mode in control participants paired with the FES sample was identified between CSA/SV neuroanatomic features and cognitive domains (LV-S2: sCCA r=-0.75, p=0.001) (**Figure S11**). In LV-S2, the latent cognitive variate, composed of symbol coding test scores (weight=-0.92), verbal fluency test scores (weight=-0.32), and digit sequencing test scores (weight=-0.24), showed the higher positive cross-loadings in the right pars opercularis (cross-loading=0.37), the right amygdala (cross-loading=0.36), the left pars opercularis (cross-loading=0.35) and the left pars triangularis (cross-loading=0.32), and higher negative cross-loading in the right middle temporal gyrus (cross-loading=-0.33) and the left supramarginal gyrus (cross-loading=-0.30) (**Figure S11 – S13**).

Patient-control cross-validations indicated the specificity of significant canonical modes identified in FES patients or matched controls (**Figure S14**).

Case-control analyses of neuroanatomy-cognition features in the two data sets

In each data set, case-control results in regional CT, CSA, and SV measures, as well as cognitive scores are demonstrated in **Figure S15 – S16**. Glass's delta (Δ) effect sizes, reflecting the extent of case-control differences in these features, are reported to be compared across the two data sets.

1. CT measure

Antipsychotic-treated patients showed significant widespread CT deficits relative to controls, with small-to-large effect sizes. The only exception was the significantly thicker cortex in the left caudal anterior cingulate gyrus (Δ=0.38, 95% CI of Δ=[0.12, 0.65], t=2.74, $p_{uncorrected}$=0.007, p_{FDR}=0.013). Pronounced CT deficits were mainly observed in frontotemporal cortices, where the bilateral rostral middle frontal gyri (Left: Δ=-0.90, 95% CI of Δ=[-1.17, -0.63], t=-6.46, $p_{uncorrected}$<0.001, p_{FDR}<0.001; Right: Δ=-1.06, 95% CI of Δ=[-1.34, -0.79], t=-7.49, $p_{uncorrected}$<0.001, p_{FDR}<0.001), the right superior frontal gyrus (Δ=-0.87, 95% CI of Δ=[-1.14, -0.59], t=-6.81, $p_{uncorrected}$<0.001, p_{FDR}<0.001), and the right medial orbitofrontal cortex (Δ=-0.81, 95% CI of Δ=[-1.08, -0.54], t=-5.62, $p_{uncorrected}$<0.001, p_{FDR}<0.001) were markedly affected.

In the FES sample, there were small-to-medium effect sizes of CT differences relative to controls, but only three regions survived FDR corrections. These regions were the left fusiform

gyrus (Δ=0.12, 95% CI of Δ=[-0.25, 0.48], t=-3.93, $p_{uncorrected}$<0.001, p_{FDR}=0.010) and bilateral precentral gyri (Left: Δ=-0.31, 95% CI of Δ=[-0.68, 0.06], t=-3.31, $p_{uncorrected}$=0.001, p_{FDR}=0.029; Right: Δ=-0.15, 95% CI of Δ=[-0.51, 0.22], t=-3.30, $p_{uncorrected}$=0.029, p_{FDR}=0.029).

2. CSA measure

It was found that only patients who received antipsychotic medications displayed significant CSA deficits when compared to controls. These effect sizes were small to large in these patients, and the regions that were most affected were mainly in the prefrontal, temporal, and parietal cortices, such as the left lateral orbitofrontal cortex (OFC) (Δ=-0.86, 95% CI of Δ=[-1.13, -0.59], t=-6.53, $p_{uncorrected}$<0.001, p_{FDR}<0.001), bilateral rostral middle frontal gyri (Left: Δ=-0.74, 95% CI of Δ=[-1.01, -0.47], t=-5.54, $p_{uncorrected}$<0.001, p_{FDR}<0.001; Right: Δ=-0.77, 95% CI of Δ=[-1.04, -0.50], t=-5.26, $p_{uncorrected}$<0.001, p_{FDR}<0.001), left banks of superior temporal sulcus (Δ=-0.77, 95% CI of Δ=[-1.04, -0.50], t=-5.68, $p_{uncorrected}$<0.001, p_{FDR}<0.001).

In the FES sample, there were small-to-medium effect sizes of CSA differences relative to controls, but no significant alterations in CSA were observed. However, the FES patients did show trends of smaller CSA in the left insula cortex (Δ=-0.58, 95% CI of Δ=[-0.95, -0.20], t=-3.34, $p_{uncorrected}$=0.001, p_{FDR}=0.076) and left transverse temporal gyrus (Δ=-0.34, 95% CI of Δ=[-0.70, 0.03], t=-2.07, $p_{uncorrected}$=0.040, p_{FDR}=0.876), although neither of these regions survived in FDR corrections.

3. SV measure

It was observed that only antipsychotic-treated patients exhibited significant subcortical alterations. It is noteworthy that increased volumes in the right pallidum can be observed in these patients (Left: Δ=0.12, 95% CI of Δ=[-0.14, 0.38], t=0.68, $p_{uncorrected}$=0.497, p_{FDR}=0.497; Right: Δ=0.39, 95% CI of Δ=[0.13, 0.65], t=2.59, $p_{uncorrected}$=0.010, p_{FDR}=0.021).

However, in the FES sample, there were trends of decreased pallidum volumes, but the results were not statistically significant (Left: Δ=-0.26, 95% CI of Δ=[-0.63, 0.11], t=-1.30, $p_{uncorrected}$=0.197, p_{FDR}=0.307; Right: Δ=-0.26, 95% CI of Δ=[-0.63, 0.11], t=-1.41, $p_{uncorrected}$=0.161, p_{FDR}=0.282).

4. Cognitive test scores

It was observed that only antipsychotic-treated patients had significant deficits in Tower of London (Δ=-1.15, 95% CI of Δ=[-1.54, -0.76], t=-4.71, $p_{uncorrected}$<0.001, p_{FDR}<0.001) and digit sequencing (digit sequencing: Δ=-0.78, 95% CI of Δ=[-1.16, -0.40], t=-4.38, $p_{uncorrected}$<0.001, p_{FDR}<0.001) test scores. This is contrary to the findings of the FES sample (Tower of London: Δ=-0.49, 95% CI of Δ=[-0.87, -0.11], t=-1.47, $p_{uncorrected}$=0.146, p_{FDR}=0.171; digit sequencing: Δ=-0.23, 95% CI of Δ=[-0.61, 0.14], t=-1.03, $p_{uncorrected}$=0.307, p_{FDR}=0.307).

Both the patients treated with antipsychotic medication and the FES sample showed significant impairments in verbal memory, token motor, and symbol coding test scores, as well as composite scores. The extent of these deficits was more pronounced in antipsychotic-treated patients compared to FES sample, except for verbal memory test scores (verbal memory: [antipsychotic-treated patients: Δ=-0.57, 95% CI of Δ=[-0.94, -0.19], t=-3.07, $p_{uncorrected}$=0.003, p_{FDR}=0.004], [verbal memory in the FES sample: Δ=-0.64, 95% CI of Δ=[-1.02, -0.26], t=-2.92, $p_{uncorrected}$=0.004, p_{FDR}=0.008]).

Supplemental Figures

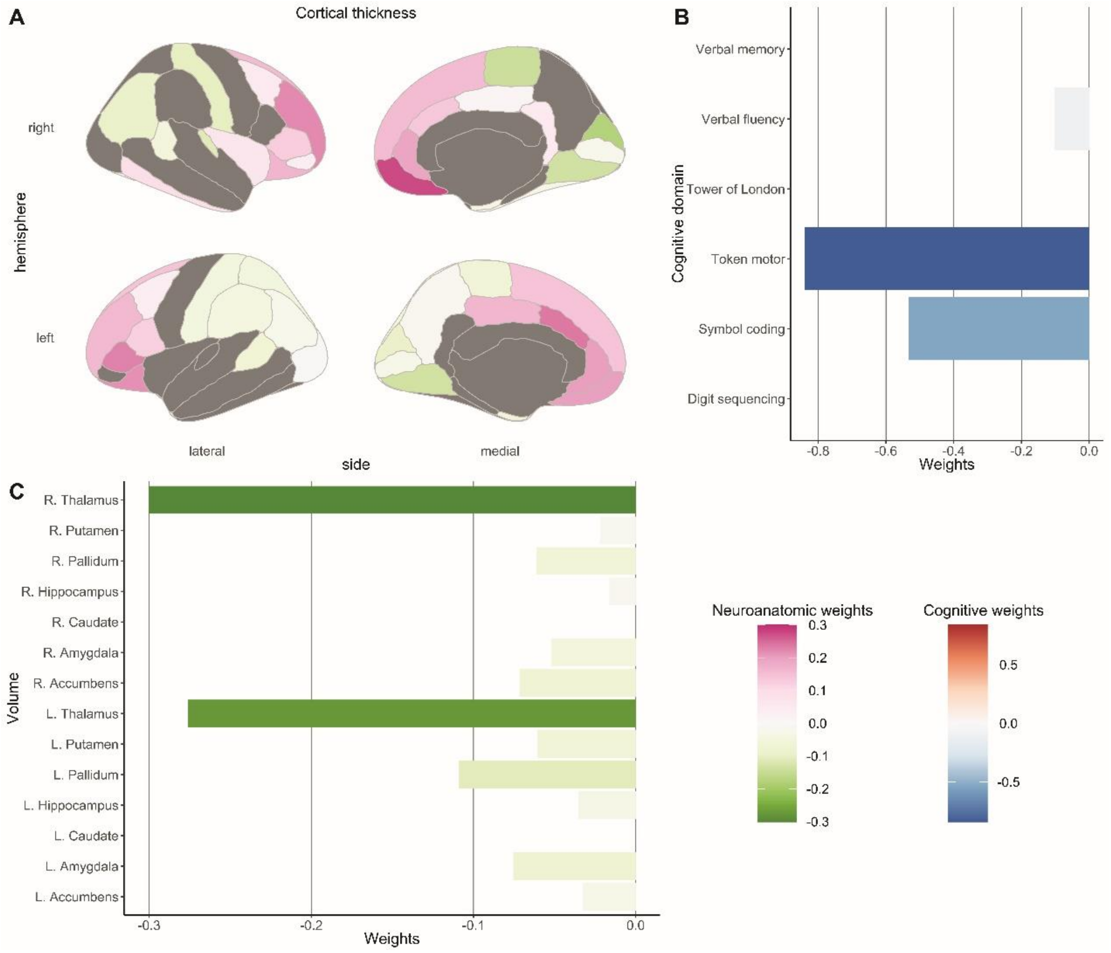

Figure S1. Non-zero weights of item variables for the significant pairs of latent variates identified in the drug-naïve sample with FES

We identified a significant canonical mode between CT/SV neuroanatomic features and cognitive domains in drug-naïve individuals with FES using an sCCA algorithm. Item variables with low contributions were penalized by regularization. In contrast, other item variables that had non-zero weights were extracted to demonstrate the composition of the significant pairs of latent variates.

Non-zero weights for (**A**) CT item variables, (**B**) cognitive item variables, as well as (**C**) SV item variables, are demonstrated for the significant pairs of latent variables in drug-naïve individuals with FES.

CT, cortical thickness; FES, first-episode schizophrenia; L, the left hemisphere; R, the right hemisphere; sCCA, sparse canonical correlation analysis; SV, subcortical volume.

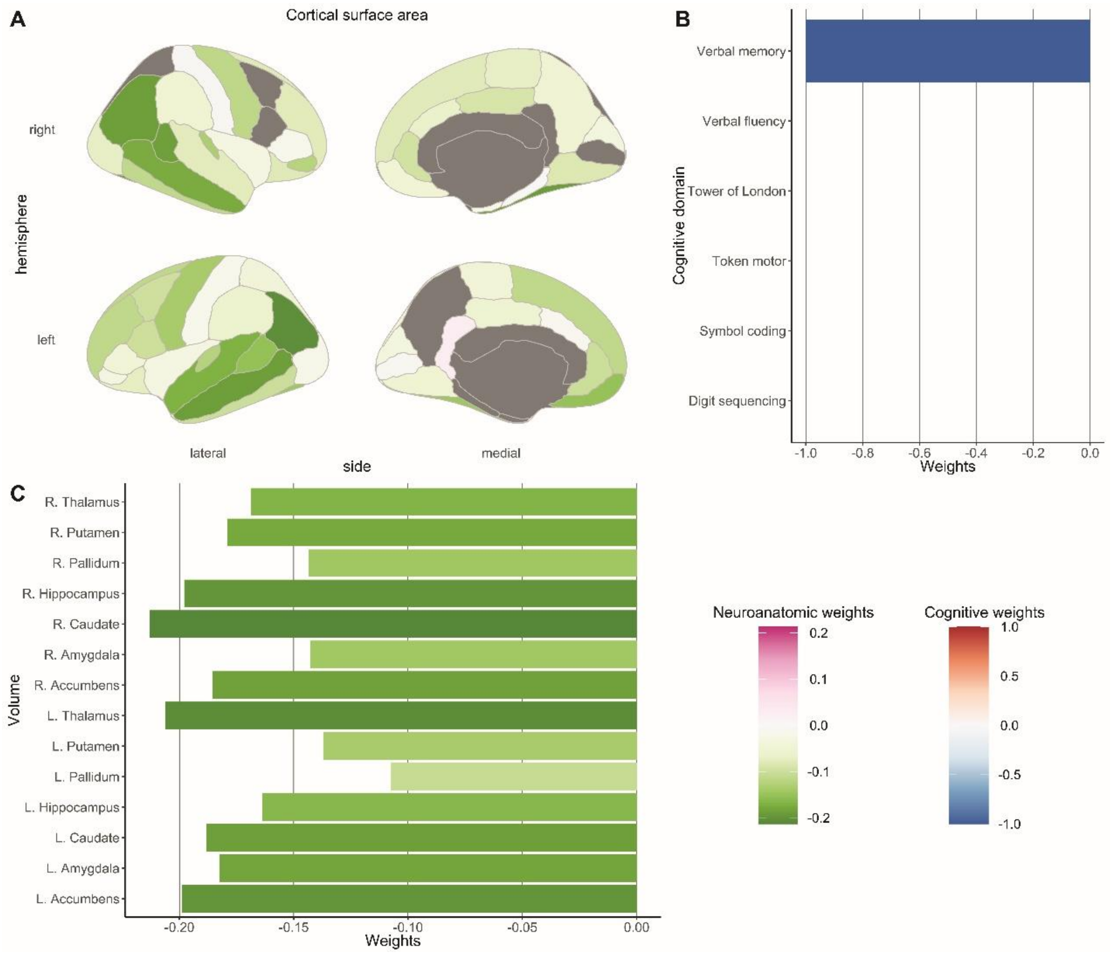

Figure S2. Non-zero weights of item variables for the first significant pairs of latent variates (LV-1) identified in the antipsychotic-treated sample

We identified two significant canonical modes between CSA/SV neuroanatomic features and cognitive domains in antipsychotic-treated individuals with schizophrenia using an sCCA algorithm. We demonstrated the first one in this figure. For each significant canonical model, item variables with low contributions were penalized by regularization. In contrast, other item variables that had non-zero weights were extracted to demonstrate the composition of the significant pairs of latent variates.

Non-zero weights for (**A**) CSA item variables, (**B**) cognitive item variables, as well as (**C**) SV item variables, are demonstrated for the first significant pairs of latent variables in antipsychotic-treated individuals with schizophrenia.

CSA, cortical surface area; L, the left hemisphere; LV-1, latent-variable-1; R, the right hemisphere; sCCA, sparse canonical correlation analysis; SV, subcortical volume.

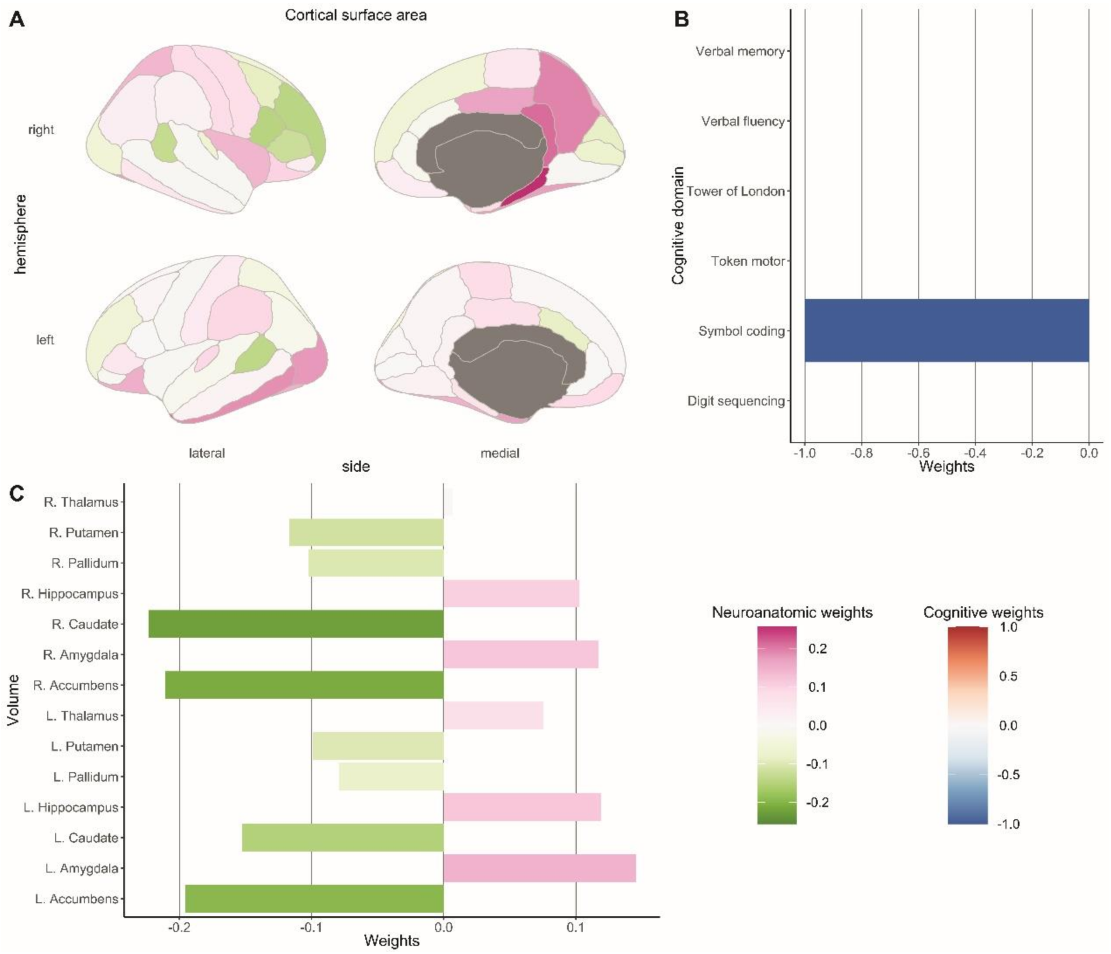

Figure S3. Non-zero weights of item variables for the second significant pairs of latent variates (LV-2) identified in the antipsychotic-treated sample

We identified two significant canonical modes between CSA/SV neuroanatomic features and cognitive domains in antipsychotic-treated individuals with schizophrenia using an sCCA algorithm. We demonstrated the second one in this figure. For each significant canonical model, item variables with low contributions were penalized by regularization. In contrast, other item variables that had non-zero weights were extracted to demonstrate the composition of the significant pairs of latent variates.

Non-zero weights for (**A**) CSA item variables, (**B**) cognitive item variables, as well as (**C**) SV item variables, are demonstrated for the second significant pairs of latent variables in antipsychotic-treated individuals with schizophrenia.

CSA, cortical surface area; L, the left hemisphere; LV-2, latent-variable-2; R, the right hemisphere; sCCA, sparse canonical correlation analysis; SV, subcortical volume.

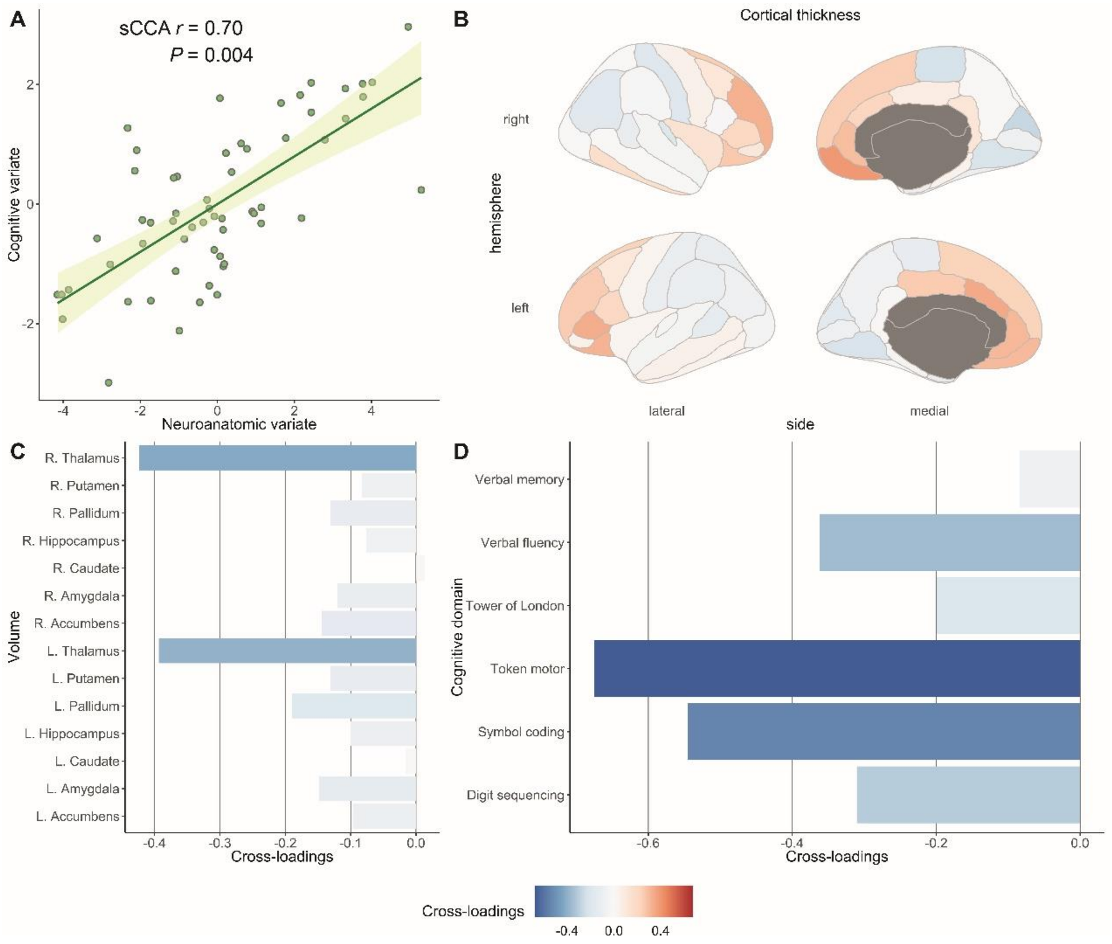

Figure S4. Cross-loadings for the significant brain-behavior canonical mode in the drug-naïve FES sample without the restriction of threshold

(**A**) We identified a significant canonical mode between CT/SV neuroanatomic features and cognitive domains in drug-naïve individuals with FES using an sCCA algorithm. Cross-loadings, representing correlations between each latent variate and the opposite item variables, were extracted without the application of threshold. We demonstrate cross-loadings between (**B**) the cognitive variate and CT item variables, (**C**) between the cognitive variate and SV item variables, and (**D**) between the neuroanatomic variate and cognitive item variables, for the significant canonical mode in drug-naïve individuals with schizophrenia.

CT, cortical thickness; FES, first-episode schizophrenia; L, the left hemisphere; p, p-value generated in the permutation test; R, the right hemisphere; sCCA, sparse canonical correlation analysis; sCCA r, the coefficient for the significant canonical correlation; SV, subcortical volume.

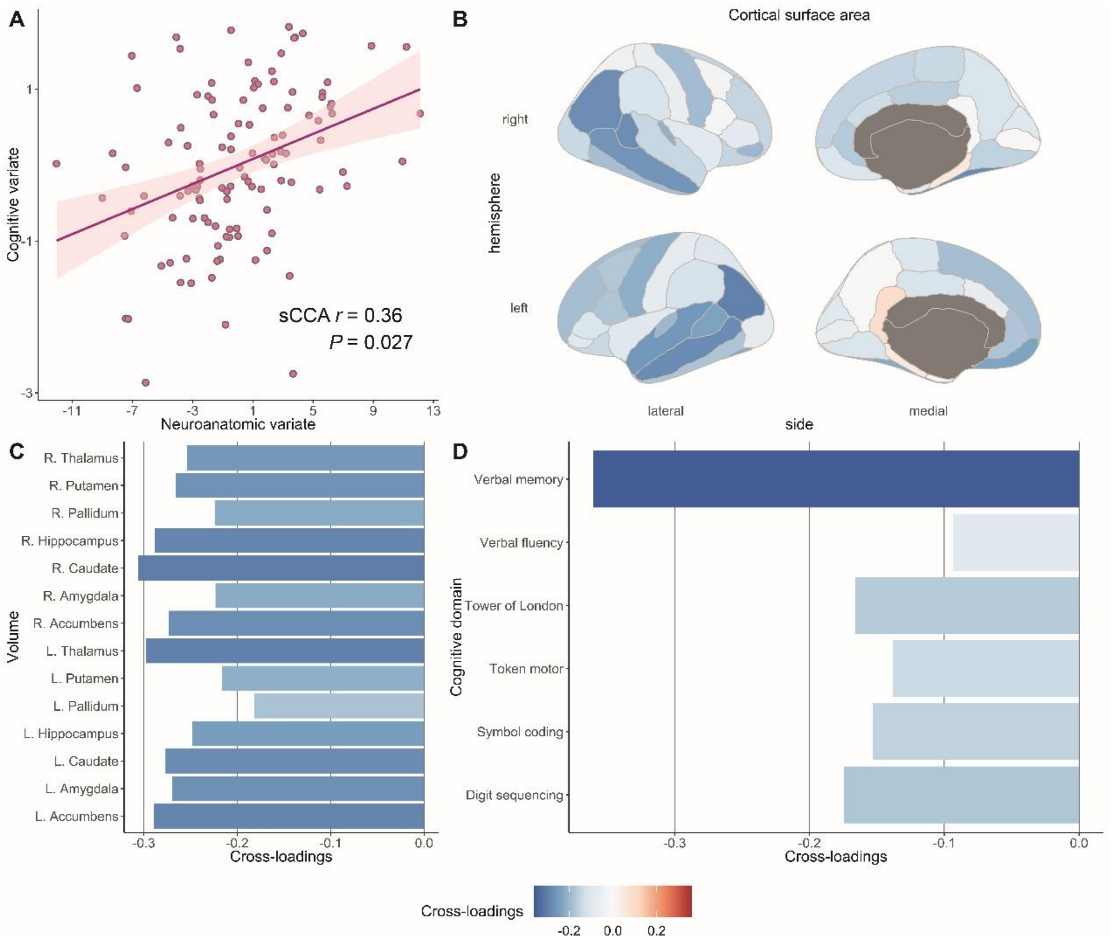

Figure S5. Cross-loadings for the first significant brain-behavior canonical mode in the antipsychotic-treated sample without the restriction of threshold

(**A**) We identified two significant canonical modes between CSA/SV neuroanatomic features and cognitive domains in antipsychotic-treated patients using an sCCA algorithm. We demonstrated the first one in this figure. Cross-loadings, representing correlations between each latent variate and the opposite item variables, were extracted without the application of threshold. We demonstrate cross-loadings between (**B**) the cognitive variate and CSA item variables, (**C**) between the cognitive variate and SV item variables, and (**D**) between the neuroanatomic variate and cognitive item variables, for the first significant canonical mode in antipsychotic-treated individuals with schizophrenia.

CSA, cortical surface area; L, the left hemisphere; p, p-value generated in the permutation test; R, the right hemisphere; sCCA, sparse canonical correlation analysis; sCCA r, the coefficient for the significant canonical correlation; SV, subcortical volume.

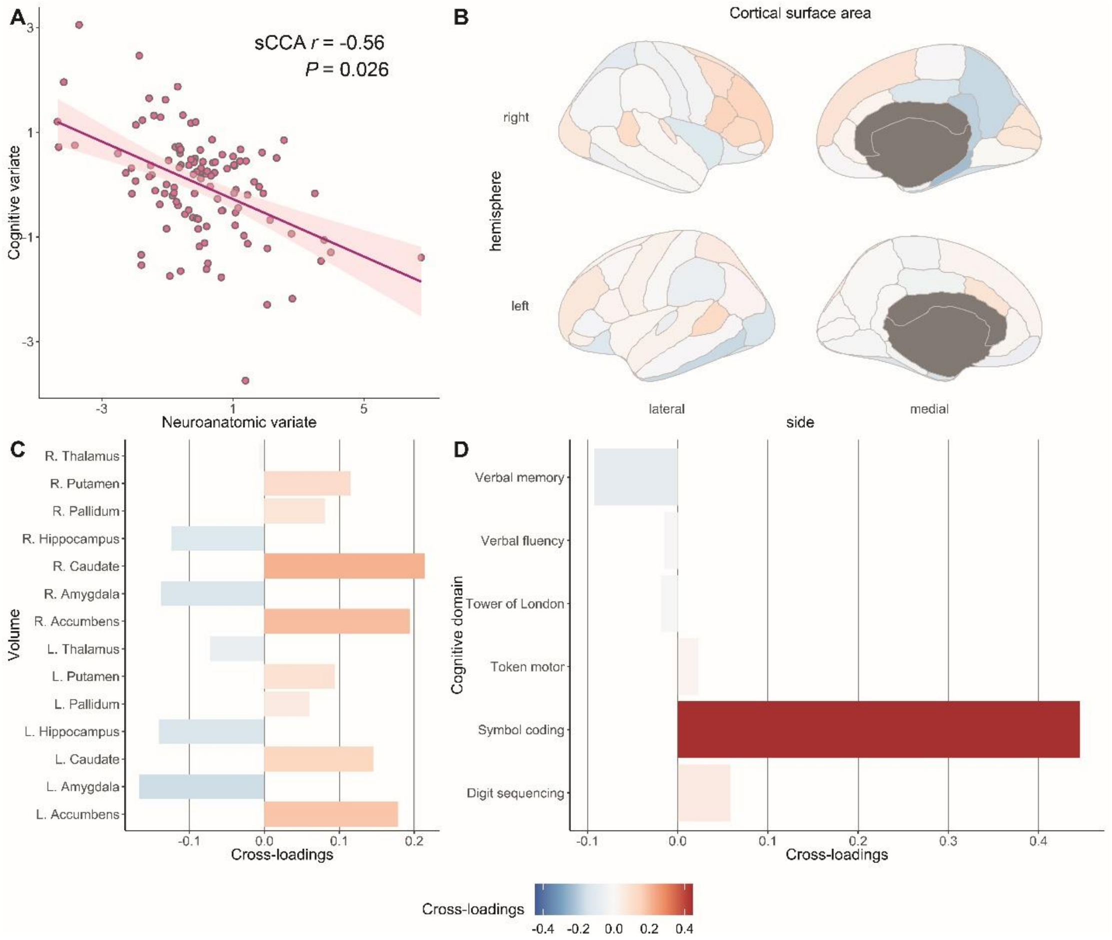

Figure S6. Cross-loadings for the second significant brain-behavior canonical mode in the antipsychotic-treated sample without the restriction of threshold

(**A**) We identified two significant canonical modes between CSA/SV neuroanatomic features and cognitive domains in antipsychotic-treated individuals with schizophrenia using an sCCA algorithm. We demonstrated the second one in this figure. Cross-loadings, representing correlations between each latent variate and the opposite item variables, were extracted without the application of threshold. We demonstrate cross-loadings between (**B**) the cognitive variate and CSA item variables, (**C**) between the cognitive variate and SV item variables, and (**D**) between the neuroanatomic variate and cognitive item variables, for the second significant canonical mode in antipsychotic-treated individuals with schizophrenia.

CSA, cortical surface area; L, the left hemisphere; p, p-value generated in permutation tests; R, the right hemisphere; sCCA, sparse canonical correlation analysis; sCCA r, the coefficient for the significant canonical correlation; SV, subcortical volume.

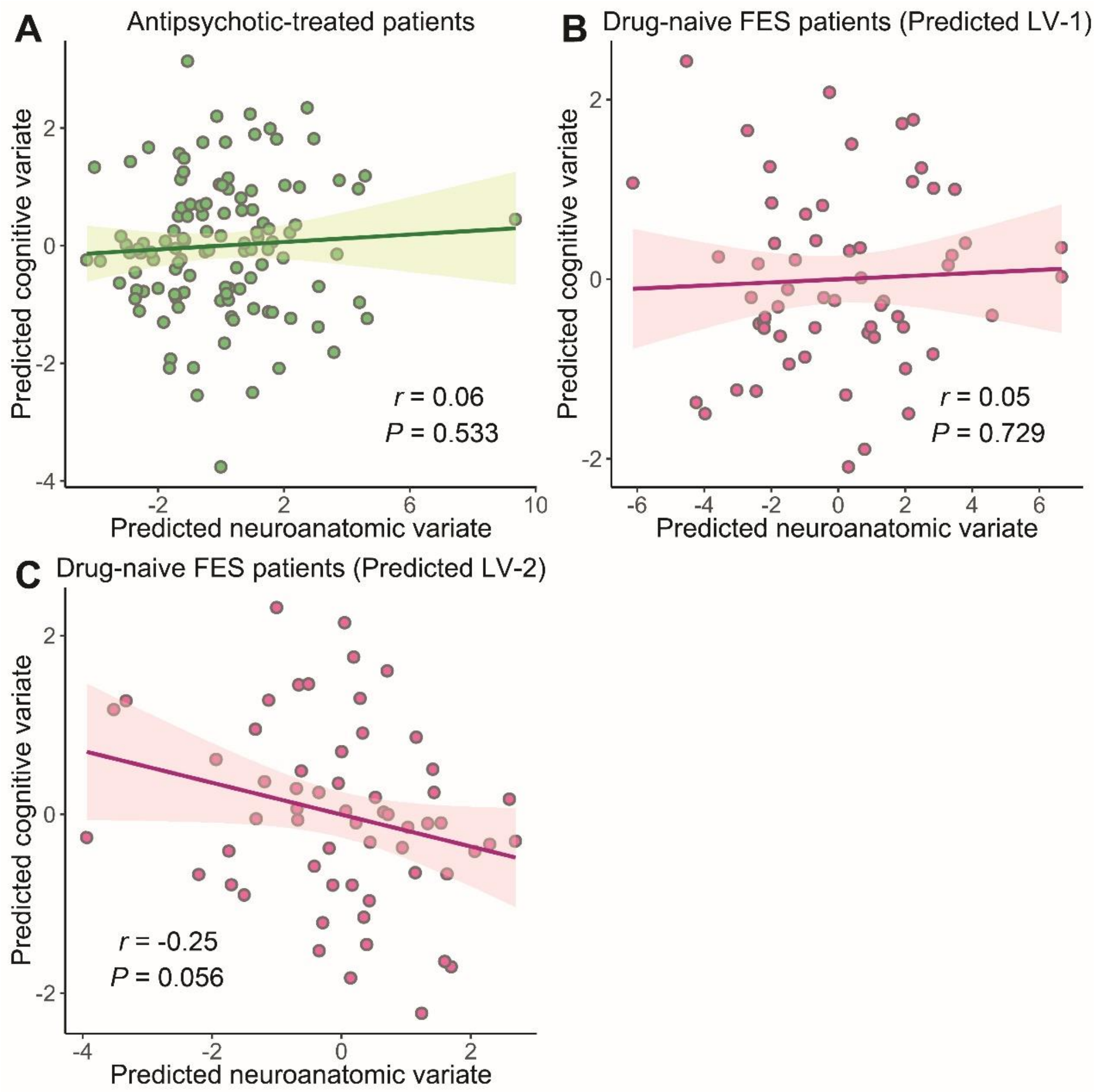

Figure S7. Test of specificity for the identified canonical modes in two samples with schizophrenia

The specificity of identified significant canonical modes was tested by validating them by crossing two samples. For a certain significant canonical mode identified in one sample, corresponding canonical weights were extracted to generate predicted latent variables in the other sample. Univariate correlation analysis was performed to test the significance of associations between predicted latent variables. The specificity of the significant canonical mode in a certain sample was defined as the non-significant univariate correlation revealed by predicated latent variables in the other sample. Non-significant univariate correlations (**A – C**) confirmed the specificity of our main findings.

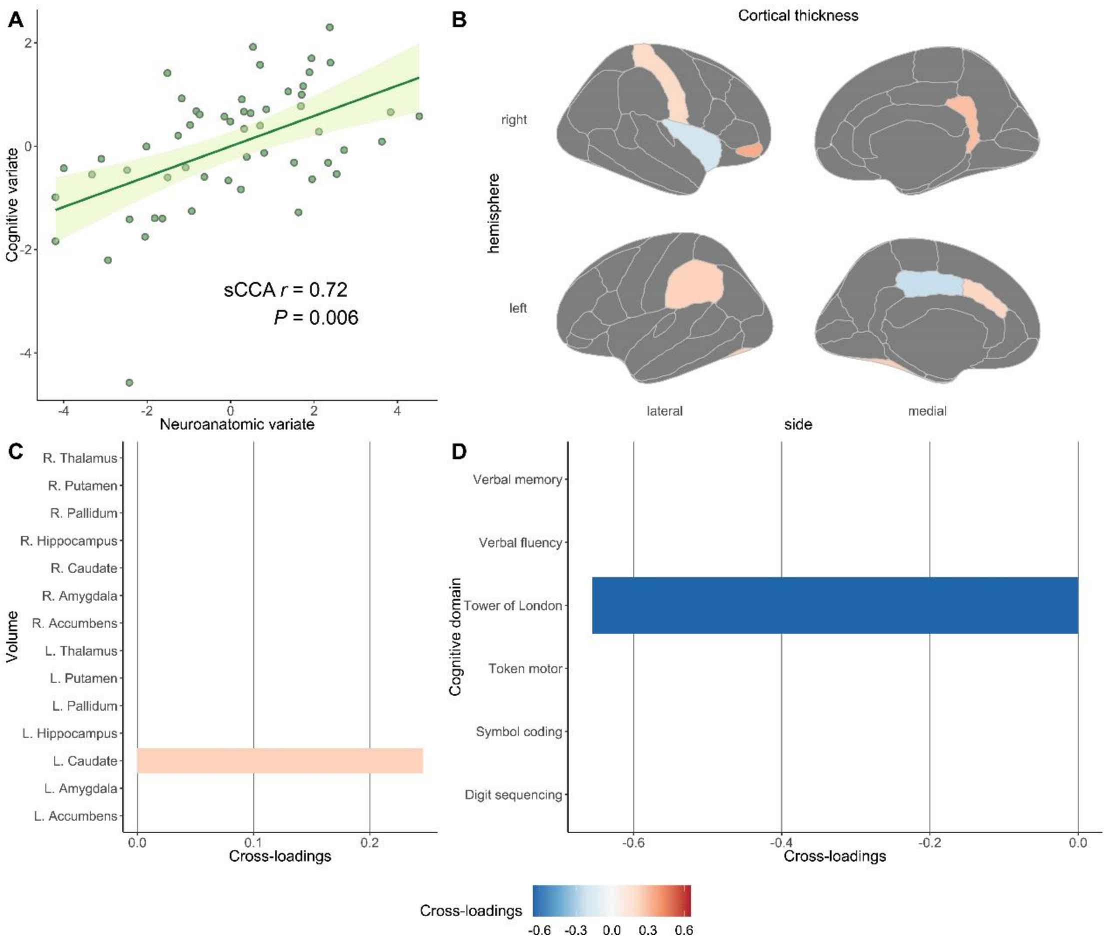

Figure S8. The first significant canonical mode (LV-S1) between brain-behavior profiles and corresponding cross-loadings with the threshold at ±0.20 in the control participants paired with the FES sample

(A) We identified a significant canonical mode between CT/SV neuroanatomic features and cognitive domains in the control participants paired with the FES sample using an sCCA algorithm. Cross-loadings, representing univariate correlation coefficients between each latent variate and the opposite item variables, were extracted at a threshold of ±0.20. We demonstrate cross-loadings between (B) the cognitive variate and CT item variables, (C) between the cognitive variate and SV item variables, and (D) between the neuroanatomic variate and cognitive item variables, for the significant canonical mode identified in the control participants paired to the FES sample.

CT, cortical thickness; L, the left hemisphere; FES, first-episode with schizophrenia; p, p-value generated in the permutation test; R, the right hemisphere; sCCA, sparse canonical correlation analysis; sCCA r, the coefficient for the significant canonical correlation; SV, subcortical volume.

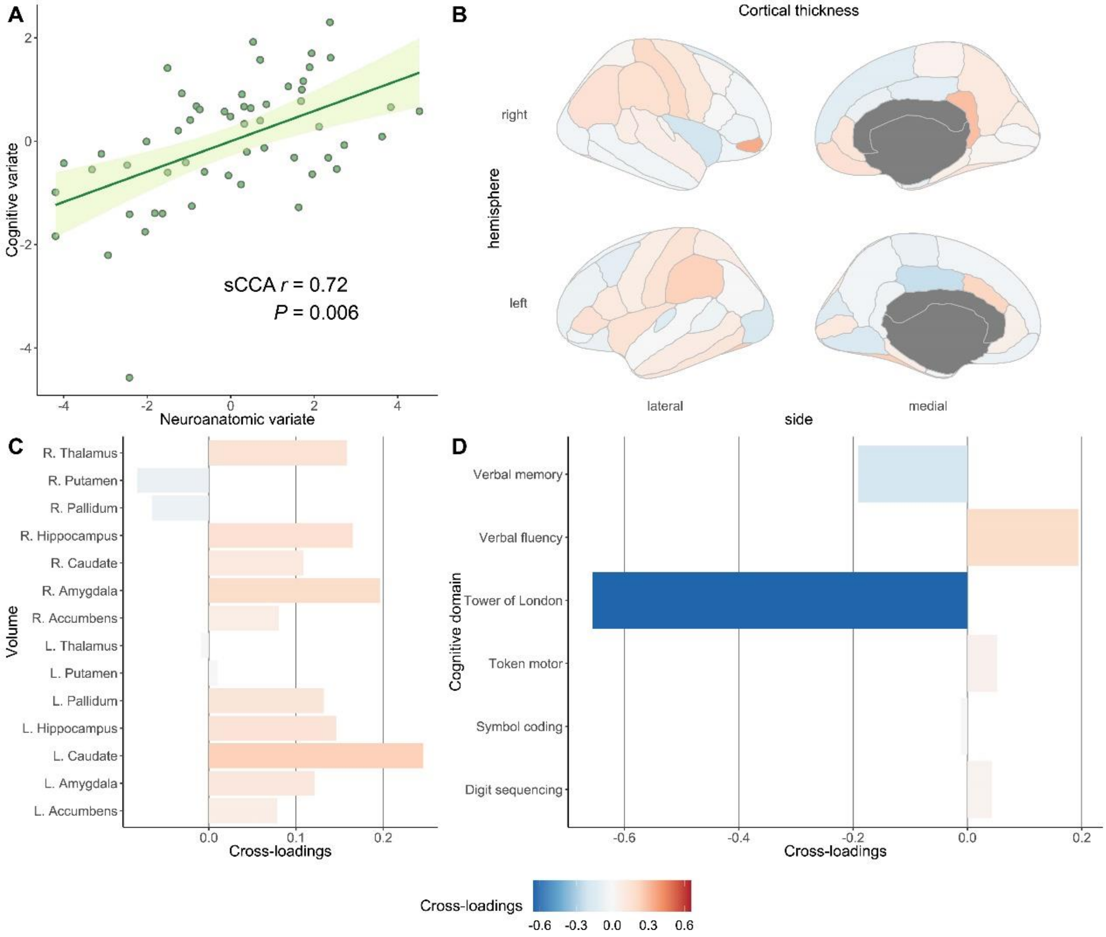

Figure S9. Cross-loadings for the first significant brain-behavior canonical mode in the control participants paired with the FES sample without the restriction of threshold

(**A**) We identified a significant canonical mode between CT/SV neuroanatomic features and cognitive domains in the control participants paired with the FES sample using an sCCA algorithm. Cross-loadings, representing correlations between each latent variate and the opposite item variables, were extracted without the application of threshold. We demonstrate cross-loadings between (**B**) the cognitive variate and CT item variables, (**C**) between the cognitive variate and SV item variables, and (**D**) between the neuroanatomic variate and cognitive item variables, for the significant canonical mode identified in the control participants paired to the FES sample.

CT, cortical thickness; FES, first-episode schizophrenia; L, the left hemisphere; p, p-value generated in the permutation test; R, the right hemisphere; sCCA, sparse canonical correlation analysis; sCCA r, the coefficient for the significant canonical correlation; SV, subcortical volume.

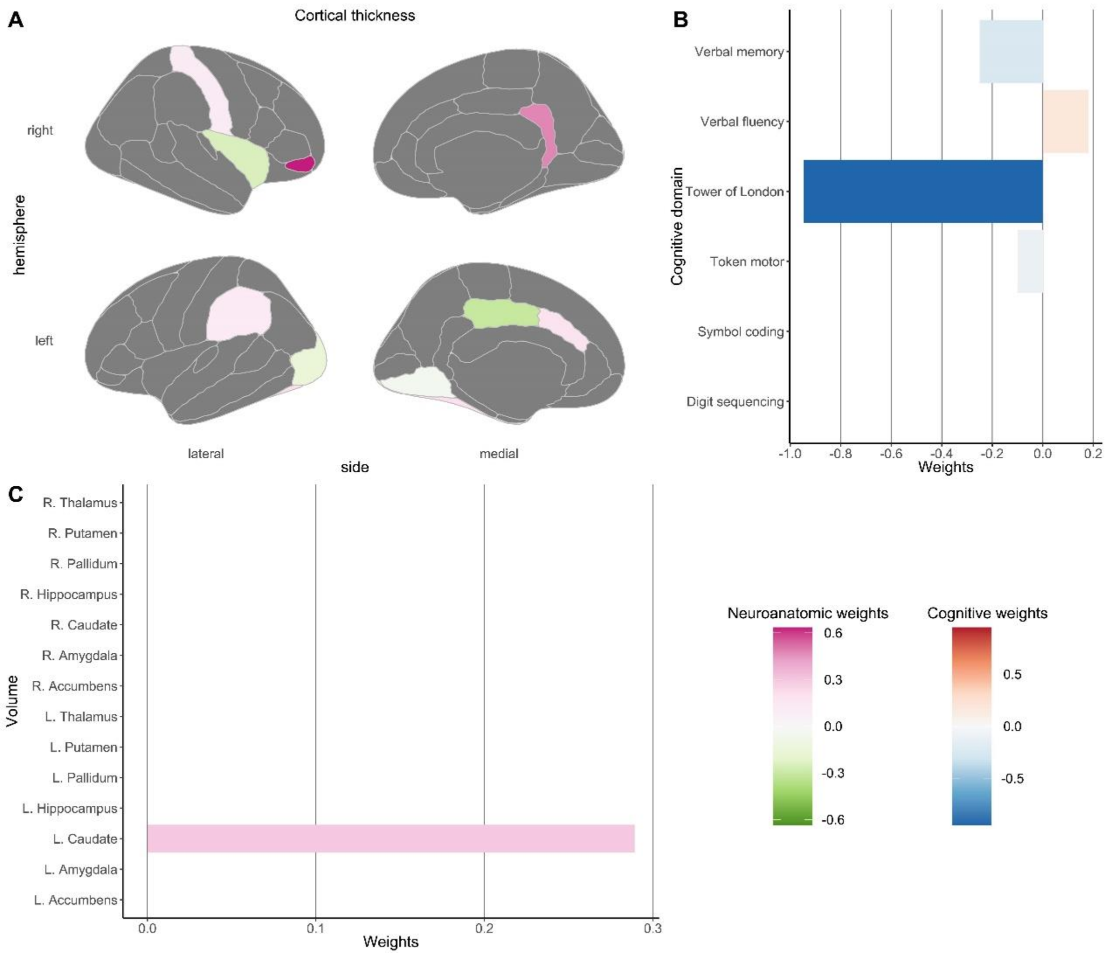

Figure S10. Non-zero weights of item variables for the first significant pairs of latent variates (LV-S1) identified in the control participants paired with the FES sample

We identified a significant canonical mode between CT/SV neuroanatomic features and cognitive domains in the control participants paired with the FES sample using an sCCA algorithm. Item variables with low contributions were penalized by regularization. In contrast, other item variables that had non-zero weights were extracted to demonstrate the composition of the significant pairs of latent variates.

Non-zero weights for (**A**) CT item variables, (**B**) cognitive item variables, as well as (**C**) SV item variables, are demonstrated for the significant pairs of latent variables in the control participants paired to the FES sample.

CT, cortical thickness; FES, first-episode schizophrenia; L, the left hemisphere; R, the right hemisphere; sCCA, sparse canonical correlation analysis; SV, subcortical volume.

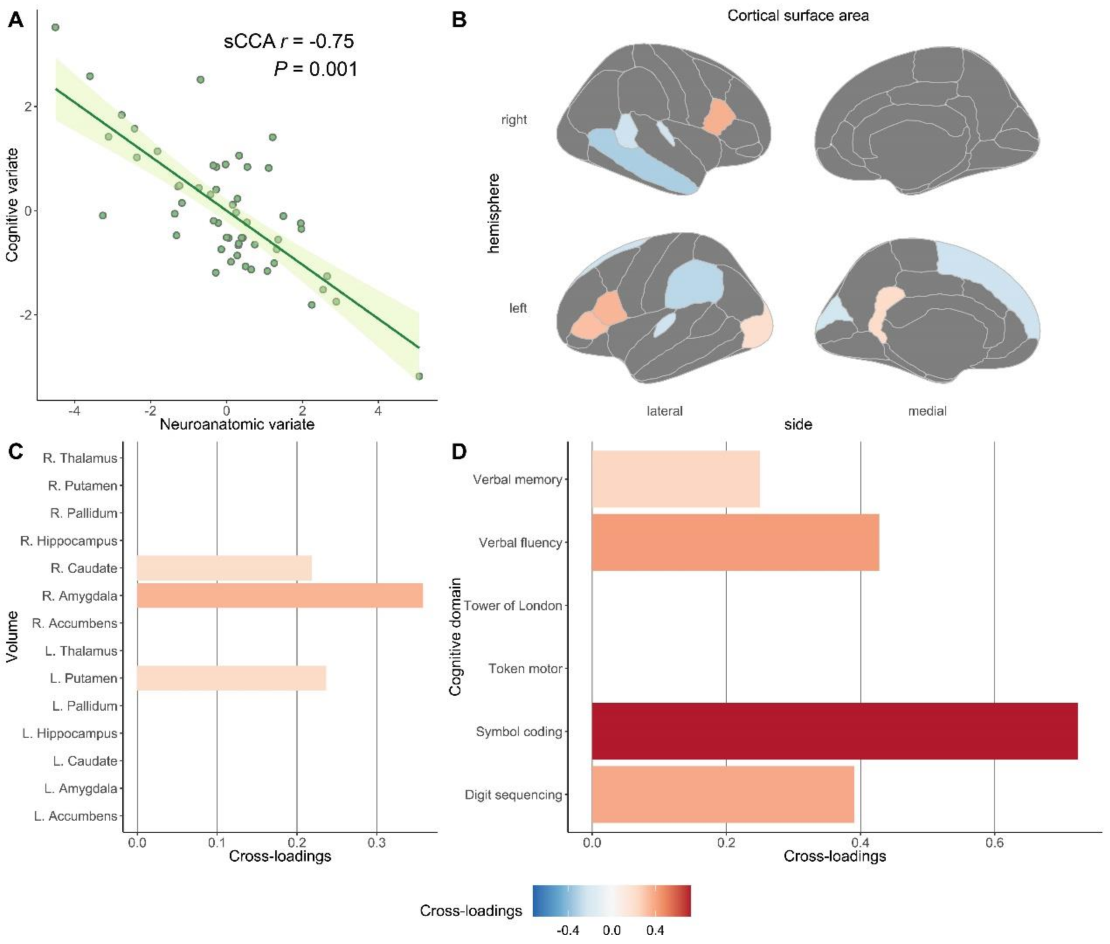

Figure S11. The second significant canonical mode (LV-S2) between brain-behavior profiles and corresponding cross-loadings with the threshold at ±0.20 in the control participants paired with the FES sample

(**A**) We identified a significant canonical mode between CSA/SV neuroanatomic features and cognitive domains in the control participants paired with the FES sample using an sCCA algorithm. Cross-loadings, representing univariate correlation coefficients between each latent variate and the opposite item variables, were extracted at a threshold of ±0.20. We demonstrate cross-loadings between (**B**) the cognitive variate and CSA item variables, (**C**) between the cognitive variate and SV item variables, and (**D**) between the neuroanatomic variate and cognitive item variables, for the significant canonical mode identified in the control participants paired to the FES sample.

CSA, cortical surface area; L, the left hemisphere; FES, first-episode with schizophrenia; p, p-value generated in the permutation test; R, the right hemisphere; sCCA, sparse canonical correlation analysis; sCCA r, the coefficient for the significant canonical correlation; SV, subcortical volume.

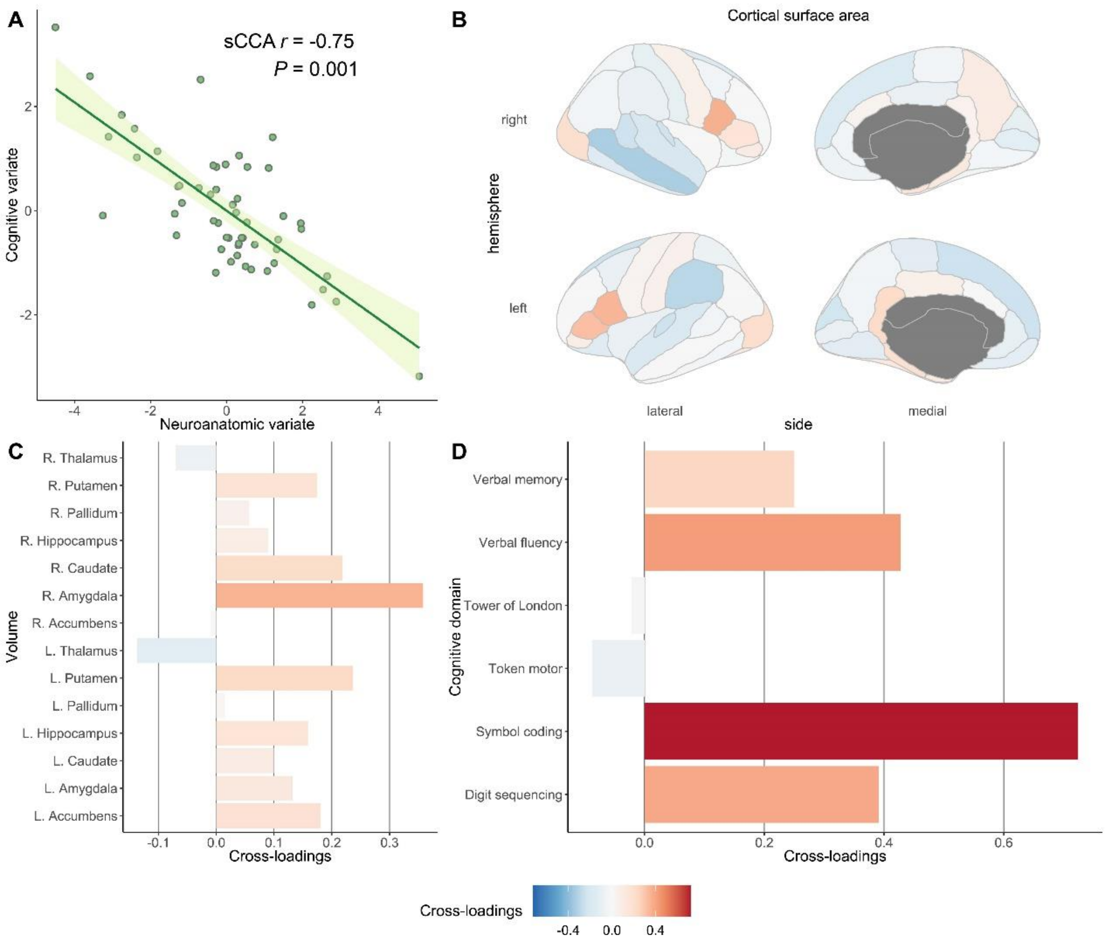

Figure S12. Cross-loadings for the second significant brain-behavior canonical mode (LV-S2) in the control participants paired with the FES sample without the restriction of threshold

(**A**) We identified a significant canonical mode between CSA/SV neuroanatomic features and cognitive domains in the control participants paired with the FES sample using an sCCA algorithm. Cross-loadings, representing correlations between each latent variate and the opposite item variables, were extracted without the application of threshold. We demonstrate cross-loadings between (**B**) the cognitive variate and CSA item variables, (**C**) between the cognitive variate and SV item variables, and (**D**) between the neuroanatomic variate and cognitive item variables, for the significant canonical mode identified in the control participants paired to the FES sample.

CSA, cortical surface area; FES, first-episode schizophrenia; L, the left hemisphere; p, p-value generated in the permutation test; R, the right hemisphere; sCCA, sparse canonical correlation analysis; sCCA r, the coefficient for the significant canonical correlation; SV, subcortical volume.

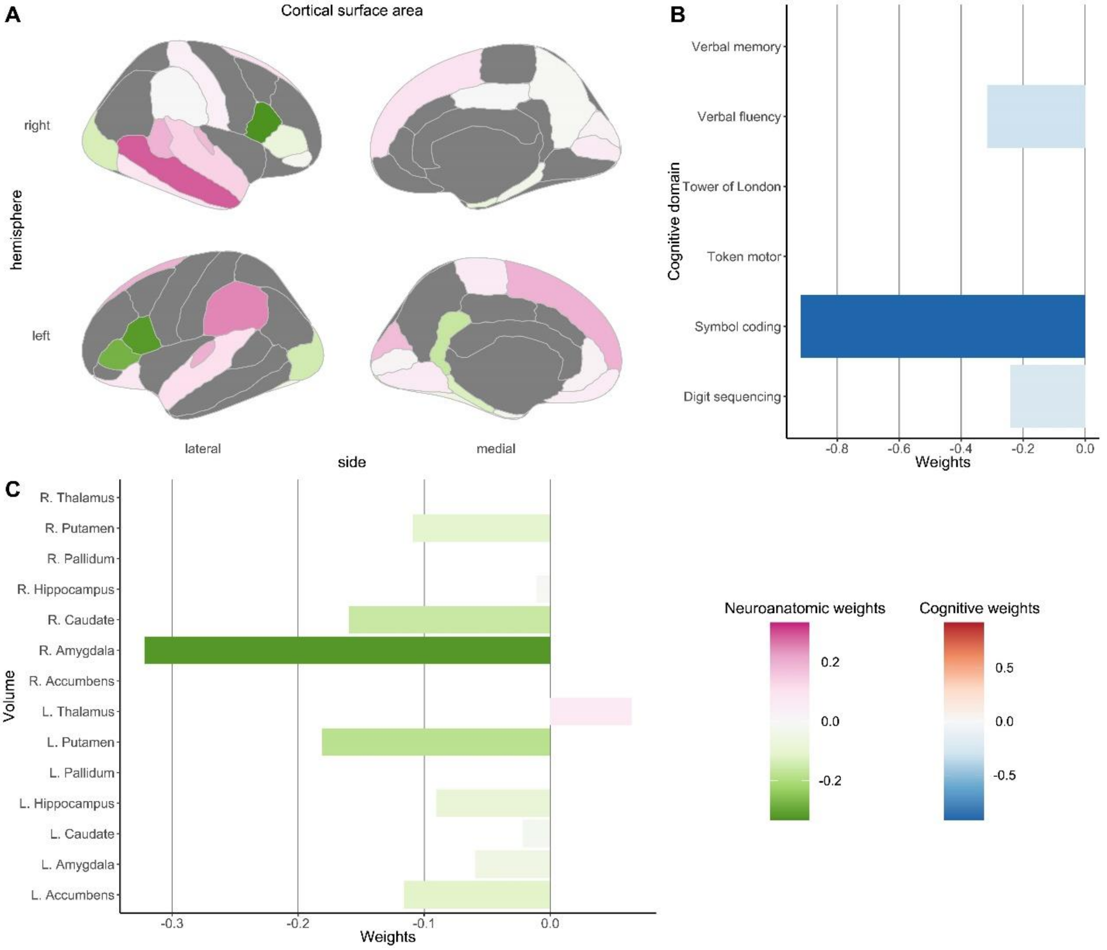

Figure S13. Non-zero weights of item variables for the second significant pairs of latent variates (LV-S2) identified in the control participants paired with the FES sample

We identified a significant canonical mode between CSA/SV neuroanatomic features and cognitive domains in the control participants paired with the FES sample using an sCCA algorithm. Item variables with low contributions were penalized by regularization. In contrast, other item variables that had non-zero weights were extracted to demonstrate the composition of the significant pairs of latent variates.

Non-zero weights for (**A**) CSA item variables, (**B**) cognitive item variables, as well as (**C**) SV item variables, are demonstrated for the significant pairs of latent variables in the control participants paired to the FES sample.

CSA, cortical surface area; FES, first-episode schizophrenia; L, the left hemisphere; R, the right hemisphere; sCCA, sparse canonical correlation analysis; SV, subcortical volume.

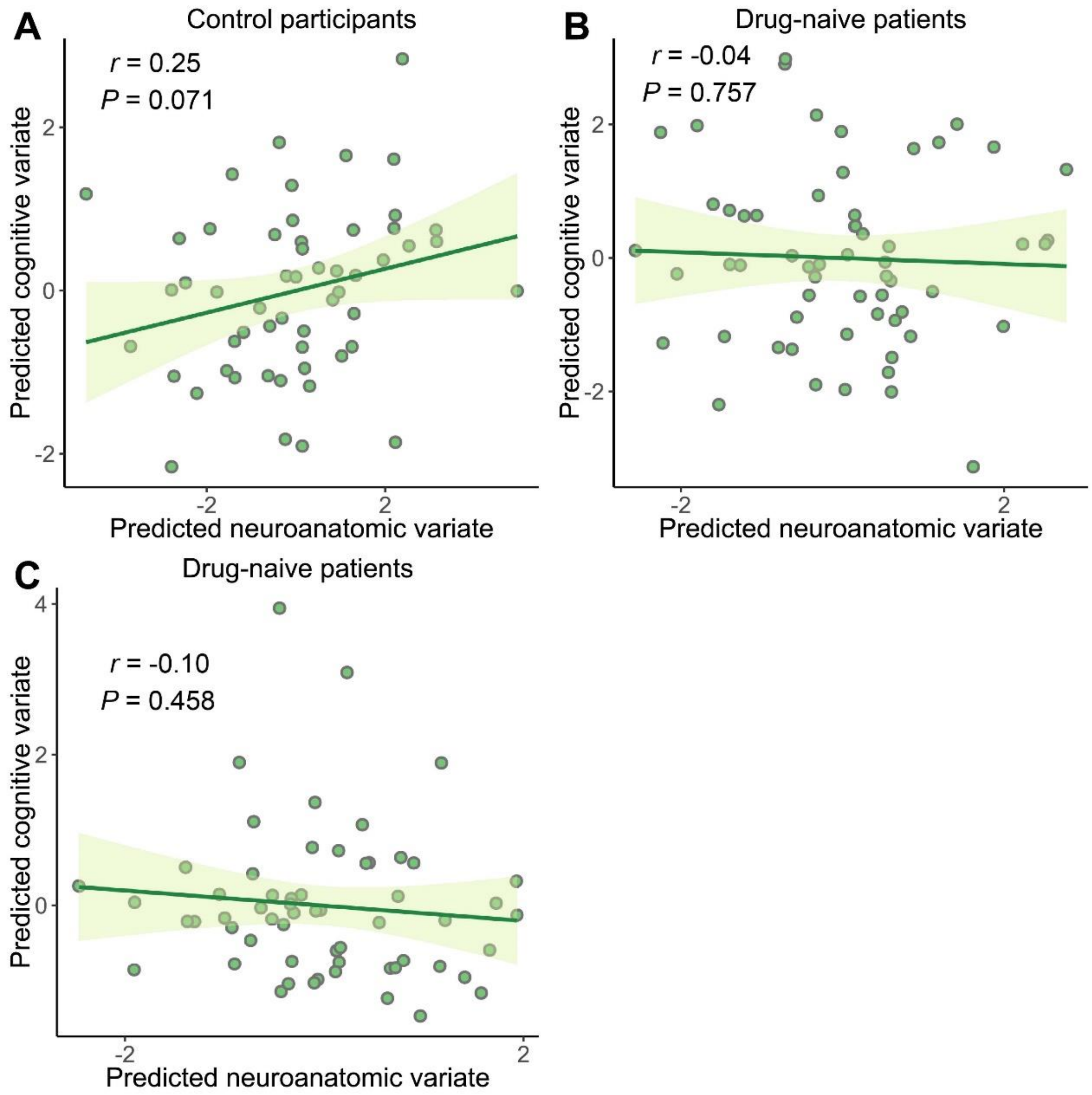

Figure S14. Test of specificity for the identified canonical modes in FES patients and matched control participants

The specificity of identified significant canonical modes was tested by validating them by crossing cases and controls. For a certain significant canonical mode identified in one sample, corresponding canonical weights were extracted to generate predicted latent variables in the other sample. Univariate correlation analysis was performed to test the significance of associations between predicted latent variables. The specificity of the significant canonical mode in a certain sample was defined as the non-significant univariate correlation revealed by predicated latent variables in the other sample. Non-significant univariate correlations (**A** – **C**) confirmed the specificity of the canonical mode identified in the FES sample.

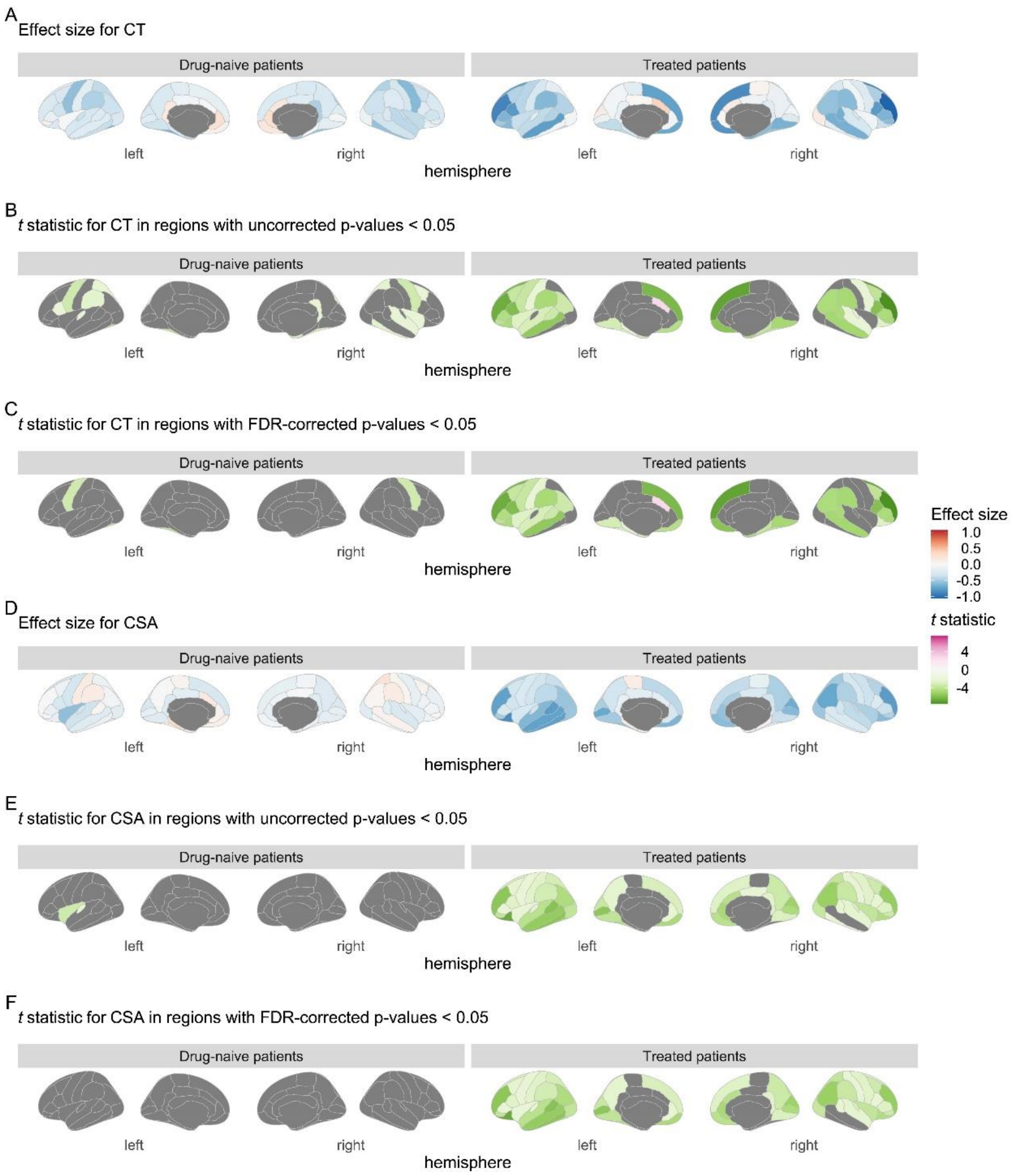

Figure S15. Cortical maps for case-control comparisons in the two data sets

We tested the significance of differences in CT and CSA between individuals with schizophrenia and corresponding matched control subjects utilizing two-sample t-tests. Glass's delta (Δ) effect sizes were also calculated to demonstrate the magnitude of case-control differences (**A**, **D**). Variance related to nuisance variables, including age and sex for CT and age, sex, and ICV for CSA, were removed before t-tests and the calculation of effect sizes. We plotted t statistics in cortical regions that survived FDR correction (**C**, **F**) and in regions with uncorrected p-values < 0.05 (**B**, **E**).

CSA, cortical surface area; CT, cortical thickness; FDR, false discovery rate; ICV, intracranial volume.

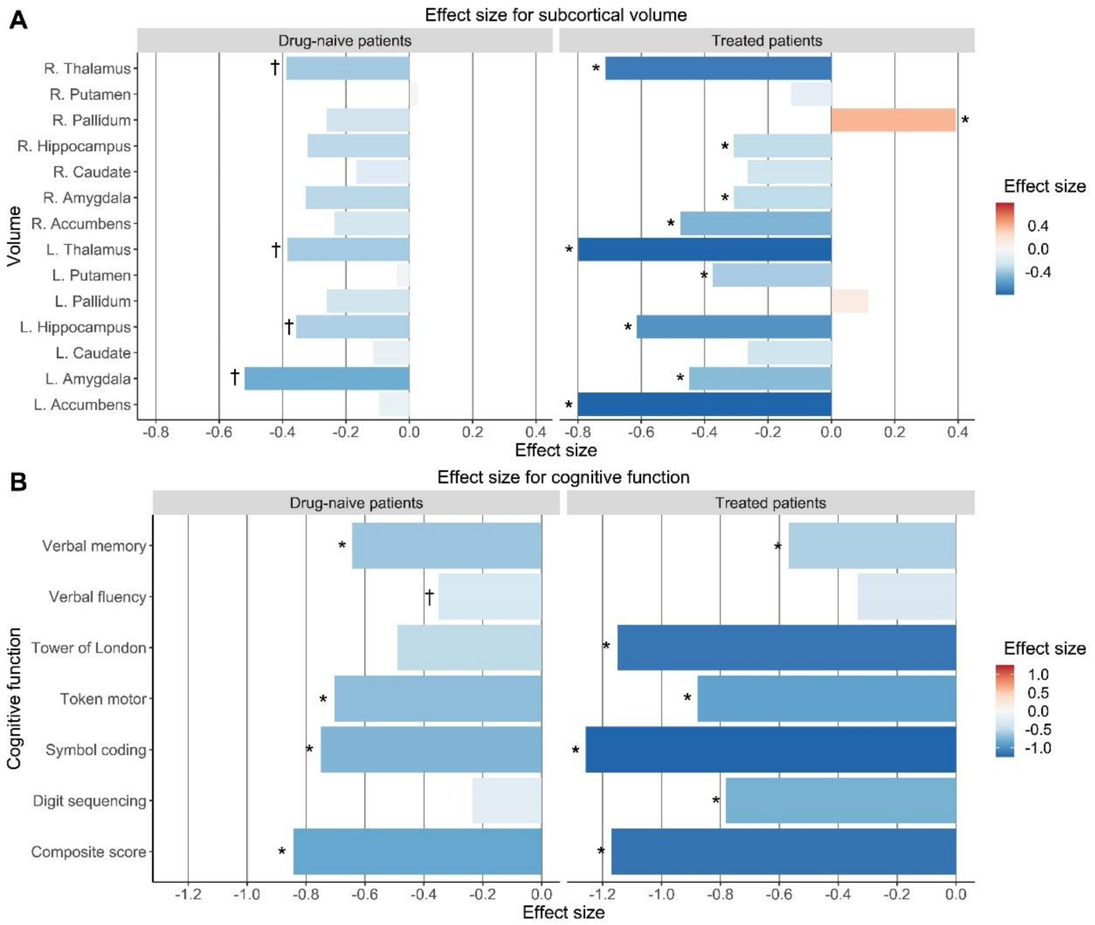

Figure S16. Case-control comparisons in SV and cognitive function in the two data sets

We tested the significance of differences in SV (**A**) and cognitive function (**B**) between individuals with schizophrenia and corresponding matched controls utilizing two-sample t-tests. Glass's delta (Δ) effect sizes were also calculated to demonstrate the magnitude of case-control differences. Variance related to nuisance variables, including age, sex, and ICV for SV and age, sex, and education level for cognitive scores, were removed before t-tests and calculating effect sizes. Asterisks (*) represent significant case-control differences that survived from FDR correction. †, uncorrected p-values < 0.05 but FDR-corrected p-values ≥ 0.05.

SV, subcortical volume; FDR, false discovery rate; ICV, intracranial volume.

Supplemental Tables

Table S1. Univariate correlations between neuroanatomic latent variables and demographics or clinical profiles in two samples with schizophrenia

	Drug-naïve sample with FES			Antipsychotic-treated sample with schizophrenia					
	CT/SV variate in LV			CSA/SV variate in LV-1			CSA/SV variate in LV-2		
Measure	r	p_{uncorr}	p_{FDR}	r	p_{uncorr}	p_{FDR}	r	p_{uncorr}	p_{FDR}
Euler number (z-score)	0.10	0.443	0.884	0.10	0.267	0.427	-0.13	0.172	0.692
Illness duration (year)	-0.17	0.187	0.884	0.22	**0.018**	0.082	-0.08	0.385	0.692
Age at onset (year)	0.06	0.663	0.884	-0.23	**0.012**	0.082	0.09	0.354	0.692
PANSS score									
Positive score	-0.15	0.267	0.884	0.06	0.538	0.691	0.13	0.185	0.692
Negative score	0.10	0.434	0.884	-0.10	0.285	0.427	0.06	0.534	0.768
General score	0.01	0.916	0.972	0.03	0.746	0.839	0.04	0.687	0.773
Total score	<0.01	0.972	0.972	-0.01	0.947	0.947	0.08	0.382	0.692
GAF score	-0.06	0.631	0.884	0.11	0.252	0.427	<0.01	0.968	0.968
CPZ equivalent (mg/day)	NA	NA	NA	0.13	0.183	0.427	-0.05	0.597	0.768

LV, the significant pair of latent variates; LV-1, the first pair of latent variates; LV-2, the second pair of latent variates; NA, not available; p_{FDR}, FDR-corrected p-value; p_{uncorr}, uncorrected p-value.

Univariate correlation analyses were conducted between neuroanatomic latent variables and demographics or clinical profiles for each significant canonical mode identified in drug-naïve individuals with FES or antipsychotic-treated individuals with schizophrenia. FDR correction was applied for generated p-values. Boldface indicates $p<0.05$.

Table S2. Univariate correlations between cognitive latent variables and demographics or clinical profiles in samples with schizophrenia

Measure	Drug-naïve sample with FES			Antipsychotic-treated sample with schizophrenia					
	Cognitive variate in LV			Cognitive variate in LV-1			Cognitive variate in LV-2		
	r	p_{uncorr}	p_{FDR}	r	p_{uncorr}	p_{FDR}	r	p_{uncorr}	p_{FDR}
Euler number (z-score)	0.08	0.558	0.580	<0.01	0.989	0.989	<0.01	0.979	0.979
Illness duration (year)	-0.30	**0.021**	0.084	0.21	**0.027**	0.123	0.07	0.445	0.910
Age at onset (year)	0.10	0.452	0.580	-0.22	**0.020**	0.123	-0.07	0.483	0.910
PANSS score									
Positive score	-0.20	0.132	0.353	0.13	0.187	0.337	-0.03	0.748	0.910
Negative score	0.32	**0.014**	0.084	0.11	0.269	0.403	0.11	0.245	0.910
General score	0.07	0.580	0.580	0.13	0.161	0.337	0.05	0.571	0.910
Total score	0.11	0.397	0.580	0.14	0.134	0.337	0.06	0.552	0.910
GAF score	-0.11	0.415	0.580	0.02	0.852	0.959	-0.02	0.809	0.910
CPZ equivalent (mg/day)	NA	NA	NA	-0.07	0.496	0.638	-0.05	0.624	0.910

LV, the significant pair of latent variates; LV-1, the first pair of latent variates; LV-2, the second pair of latent variates; NA, not available; p_{FDR}, FDR-corrected p-value; p_{uncorr}, uncorrected p-value.

Univariate correlation analyses were conducted between cognitive latent variables and demographics or clinical profiles for each significant canonical mode identified in drug-naïve individuals with FES or antipsychotic-treated individuals with schizophrenia. FDR correction was applied for generated p-values. Boldface indicates p<0.05.

Table S3. Case-control comparisons in demographics within the data set

	Data set 1			
Measure	Drug-naive individuals with FES (N=59)	Matched control subjects (N=59)	t/χ^2	p-value
Age (M [SD], years)	28.46 (9.24)	24.75 (2.41)	2.98	**0.004**
Sex (Female, n/%)	31 (52.5%)	43 (72.9%)	5.22	**0.022**
Education level (M [SD], years)	11.05 (3.40)	17.34 (1.58)	-12.88	**<0.001**

	Data set 2			
Measure	Antipsychotic-treated individuals with schizophrenia (N=115)	Matched control subjects (N=115)	t/χ^2	p-value
Age (M [SD], years)	45.95 (7.10)	45.85 (7.88)	0.10	0.923
Sex (Female, n/%)	41 (35.7%)	41 (35.7%)	0.00	>0.99
Education level (M [SD], years)	10.15 (3.10)	9.90 (3.65)	0.55	0.580

FES, first-episode schizophrenia; M, mean value; SD, standard deviation; t, t statistic in the two-sample t-test; χ^2, chi-square statistic.

Demographics in the data set of drug-naïve individuals with FES and control subjects were slightly mismatched. Case-control comparisons in demographics were conducted within the data set, including two-sample t-tests for age and education level comparisons and chi-square tests for sex distribution differences. Boldface indicates p<0.05.
